# New Genus and New Subgenera of Camerobiid Mites (Acari: Prostigmata: Camerobiidae) with a Key to World Species of the Genus *Neophyllobius* †

**DOI:** 10.3390/insects13040344

**Published:** 2022-03-31

**Authors:** Jawwad Hassan Mirza, Muhammad Kamran, Fahad Jaber Alatawi

**Affiliations:** Acarology Research Laboratory, Department of Plant Protection, College of Food and Agriculture Sciences, King Saud University, Riyadh 11451, Saudi Arabia; jmirza@ksu.edu.sa (J.H.M.); kamran1513@gmail.com (M.K.)

**Keywords:** classification, ventral idiosoma, *Monobius*, *Monophyllobius*, comb. nov

## Abstract

**Simple Summary:**

The present study erects a new genus, *Monobius* Alatawi and Kamran, where all the leg tarsi in females have one midventral seta. Moreover, the genus *Tillandsobius* Bolland is synonymized with the genus *Tycherobius* Bolland and the genus *Neophyllobius* Berlese is categorized in two new subgeneric divisions. For the first time, a key to all known species of the genus *Neophyllobius* is provided. The ambiguities in the ventral idiosoma setal notation are highlighted and discussed.

**Abstract:**

A new genus, *Monobius* Alatawi and Kamran, is hereby proposed for the two already described species, viz; *M*. *electrus* (Żmudziński) and *M*. *meyerae* (Bolland). In addition, the monospecific genus *Tillandsobius* Bolland is synonymized with the genus *Tycherobius* Bolland due to variations in the setae number of tibiae I–IV. Further, the genus *Neophyllobius* Berlese is categorized in two new subgeneric divisions as *Neophyllobius* Berlese and *Monophyllobius* Mirza. The number and position of the midventral setae on tarsi I–IV are considered as strong diagnostic generic and subgeneric diagnostic characters. The present study also includes the key to all known species of the genus *Neophyllobius*. The morphological characters of ten poorly described *Neophyllobius* species were studied in detail through published literature. The ambiguities in the ventral idiosoma setal notation are highlighted and discussed. It is concluded that two intercoxal setae *3a*–*4a* are always present on small platelets, paired aggenital setae (*ag*) are present anteriorly and paired genital setae (*g*) present posteriorly on genital shield. In addition, five records of new species for Saudi Arabia are reported along with re–descriptions of three species.

## 1. Introduction

The family Camerobiidae Southcott (Acari: Prostigmata) is the second largest family in the superfamily Raphignathoidea. It consists of more than 170 species in seven genera that can be differentiated in two groups based on the position of the solenidion on tarsi I–II. The three genera, viz; *Neophyllobius* Berlese [1], *Tillandsobius* Bolland [2] and *Tycherobius* Bolland [2] have a solenidion present on the basal half of the tarsi I–II, while the four genera, *Acamerobia* Fan and Walter [3], *Camerobia* Southcott [4], *Bisetalobius* du Toit, Theron and Ueckermann [5] and *Decaphyllobius* Bolland [2] have a solenidion present on distal half of tarsi I–II.

The camerobiid mites, also known as stilt–legged mites, are non-potential predators feeding on different phytophagous pest mites and crawlers of scale insects [2]. Although they are widely distributed in both, temperate and tropical zones, their biology is not yet studied [2,3]. In the field, camerobiid mites are present in low numbers as reported for all the described species, globally [2,3,4,5]. There are four active developmental stages, viz. larva, protonymph, deutonymph and adult), which can be found on ground cover grasses, on plant foliage and under the bark of the trees [2,3,4].

*Neophyllobius* is the largest genus of the family Camerobiidae, having 139 species to date [6,7,8,9]. Berlese [1] erected the genus *Neophyllobius* with the type species *N*. *elegans* and designated it to the family Tetranychidae. Later, this genus was transferred to different families, including Stigmaeidae [10], Raphignathidae [11] and Calligonellidae [12,13], mainly due to the misinterpretations of mouthparts and stylophore. Southcott [4] identified the uniqueness of the genus, erected the new family Neophyllobiidae for *Neophyllobius* (without a camerostome and mouthparts anterior) and proposed a new family Camerobiidae for the genus *Camerobia* (with a camerostome and mouthparts inferior). Gerson [14] declared these morphologies as “misinterpretations” and synonymized Neophyllobiidae with the Camerobiidae and gave detailed diagnoses of the two genera included.

Based on the number of setae on tibiae I–IV and the position of two midventral setae on leg tarsi I–II, Bolland [2] erected two genera, *Tycherobius* and *Tillandsobius*, in the family Camerobiidae. The type species of the two genera (*T*. *lombardinii* and *Ti*. *floridensis*, respectively) were transferred from the genus *Neophyllobius* [2]. The genera *Tycherobius* and *Tillandsobius* currently include 25 and 1 species, respectively [7,15]. The systematics of the genus *Neophyllobius* was intensively studied by Bolland [16], where 50 new species were proposed, and 35 species were redescribed. Up to now, some regional keys of the genus have been published from Iran [17,18,19,20], Turkey [4,21,22] and Mexico [6]. Recently, Nasrollahi et al. [23] and Fan and Walter [3] provided the morphological characters of 25 species of *Tycherobius* and the camerobiid genera, respectively.

The ventral idiosoma chaetotaxy is mostly fixed and has less taxonomical importance [24]. However, the species of *Neophyllobius* have been distinguished based on differences in the lengths of coxal setae [16]. In this genus, the setal notation on the ventral idiosoma has been inconsistent in the literature. There are four different kinds of descriptions/illustrations present based on absence of intercoxal setae (*3a* or *4a*), the absence of aggenital setae (*ag*) and the presence of one or two pairs of genital setae (*g* or *g1*–*2*). Kethley [25] and Fan and Walter [3] made some efforts towards highlighting this confusion. However, to date, the situation remains ambiguous.

The global camerobiid mite fauna, including that of Saudi Arabia (SA), require special consideration. Previously, two camerobiid genera (*Neophyllobius* and *Decaphyllobius*) and six species (*N*. *muscantribii* Bolland, *N*. *fissus* De Leon, *N*. *hispanicus* Bolland, *N*. *gonzali* Zaher and Gomaa, *N*. *communis* Gerson, and *D*. *gersoni* Bolland) have been reported from SA [26,27]. In the present research work, a new genus; *Monobius* Alatawi and Kamran gen. nov. is proposed. The monospecific genus *Tillandsobius* is synonymized with *Tycherobius*, raising the number of species to 26 in the latter genus. The comparative morphological characters of the three genera (*Monobius*, *Neophyllobius* and *Tycherobius*) are provided. The genus *Neophyllobius* is divided into two new subgenera, *Neophyllobius* Berlese and *Monophyllobius* Mirza. The inconsistencies in the ventral idiosoma setal notation are discussed. The morphological characters of a few poorly described species of the genus *Neophyllobius* are added. The present research provides the key to the world species of the genus *Neophyllobius*. Also, five new records from Saudi Arabia including three species redescriptions were given.

## 2. Materials and Methods

The camerobiid mites were collected using two different methods: (a) shaking plant foliage on a sheet of white paper, picking the freely moving mites by a camel hair brush, and storing in 1.5 mL vials filled with 70% ethanol, (b) scooping out the soil debris and leaf litter under trees and shrubs and storing it in a labelled plastic bag. The soil debris and leaf litter samples were processed through a Berlese funnel for at least eight hours where the specimens were collected in a water filled plastic bowl placed underneath the funnel. The collected camerobiid mites from both methods were permanently slide mounted using the Hoyer’s medium under a stereomicroscope (Olympus^®^, SZX10, Tokyo Japan). The slide mounted specimens were identified under the phase contrast microscope (Olympus^®^, BX51, Tokyo, Japan). Different body parts of mites for re–descriptions and illustrations, were pictured with the Auto–montage Software System (SYNCROSCOPY^®^, Cambridge, UK) attached to a phase contrast microscope (Leica^®^, DM2500, Wetzlar, Germany). Final processing of drawings was done in Adobe Illustrator (Adobe Systems Incorporated, San Jose, CA, USA). The terminology used in this study follows that of Grandjean [28], Bolland [2] and Kethley [25]. All the measurements of morphological characters in the redescriptions are provided as ranges in micrometres. Only the published species descriptions and illustrations were used in the present study to compare morphological differences and variations. The collected specimens from Saudi Arabia were deposited at King Saud University Museum of Arthropods (KSMA, Acarology section), Department of Plant Protection, College of Food and Agriculture Sciences, King Saud University, Riyadh, Saudi Arabia.

## 3. Results

### 3.1. Family Camerobiidae Southcott, 1957

Camerobiidae Southcott, 1957: 311 [4].

Neophyllobiidae Southcott, 1957: 311, synonymized by Gerson, 1972: 507 [14].

**Type genus:** *Camerobia* Southcott, 1957: 311.

**Diagnosis:** Idiosoma ovoid or nearly circular, dorsoventrally compressed; some or all legs longer than idiosoma; free leg segments annulated, femora and tibiae longer than other leg segments; genua short often with a whip–like long setae, gnathosoma jointed to idiosoma in an inferior position; palpi weak, short, without tibial claw; peritremes in one or several loops.

### 3.2. Synonymy of the Genus Tillandsobius

Considering the genera of the family Camerobiidae up to now, three genera i.e., *Neophyllobius*, *Tillandsobius* and *Tycherobius* are differentiated based on the number of setae on tibiae I–IV and the difference in position of the midventral setae on tarsi I–II [2]. As reported in different published literature [3,23,29], we found that the number of setae on tibiae I–IV were variable and not suitable for generic differentiation (Table 1). For instance, the species *T*. *rhytis* [29] has an almost similar tibial chaetotaxy to that for the type species of the genus *Tillandsobius*, *Ti*. *floridensis* i.e., 8–7–7–7 vs. 8–7–6–6 (excluding solenidion) [2,23,29,30]. The type species of the genus *Tycherobius*, *T*. *lombardinii* (McGregor), was redescribed by Bolland [2] with tibial chaetotaxy as 9–8–7–7. However, the recent publications reported tibiae I–IV with 8–7–6–6, which is exactly similar to the tibiae I–IV setal count for *Ti*. *floridensis* [23,29]. This further represents the variation in this morphological character.

Additionally, there are two *Neophyllobius* species, *N*. *fani* Doğan and Ayyildiz [31] and *N*. *succineus* Bolland and Magowski [32], which have almost the same number of setae on tibiae I–IV as in *Ti*. *floridensis* (McGregor) [2] (8–7–7–6 vs. 8–7–6–6). Furthermore, there are three *Neophyllobius* species, *N*. *podocarpi* Bolland [16], *N*. *nemoralis* Kuznetsov and Livshits [33] and *N*. *parthenocissi* Bolland [16], which have the same number of setae on tibiae I–IV as in most of species of the genus *Tycherobius* (9–8–7–7 vs. 9–8–7–7) (Table 1) [15].

These three genera were also differentiated based on the position of midventral setae on tarsi I–II. The genus *Neophyllobius* has one or two midventral setae on tarsi I–IV, if two setae are present, they are in a longitudinal line. While the genera *Tillandsobius* and *Tycherobius* always have two midventral setae on tarsi I–II, consistently not in a longitudinal line and variously spaced (Table 1). In this aspect, the genera *Tillandsobius* (one species) and *Tycherobius* (25 species) are closely related.

Based on the evidence provided above, the number of setae on tibiae I–IV does not represent a strong morphological character to differentiate the three genera *Neophyllobius*, *Tillandsobius* and *Tycherobius*. However, the number and position of the mid–ventral setae on tarsi I–II, remain a constant and persistent generic diagnostic character. Therefore, the monospecific genus *Tillandsobius* is hereby synonymized with the genus *Tycherobius*. In addition, we propose a new genus, *Monobius* Alatawi and Kamran gen. nov., for the two species (one midventral seta on all leg tarsi along with a proximal solenidion) namely, *M*. *meyerae* (Bolland) and *M*. *electrus* (Żmudziński), described originally in the genus *Neophyllobius*. The diagnoses of the genera *Neophyllobius* and *Tycherobius* are modified and provided below. The morphological characters of these three genera, including the new genus, are summarized in Table 1.

### 3.3. New Genus Monobius Alatawi and Kamran

urn:lsid:zoobank.org:act:3B429EFF-A148-46A5-8BE4-32127A03E707

**Type species**: *Neophyllobius electrus* Żmudziński, 2020:3 [9].

**Diagnosis:** Leg tarsi I–II with one mid-ventral seta present on distal half and a proximal solenidion, tarsi I–IV with 9–9–7–7 tactile setae.

**Remarks:** The new genus *Monobius*, is morphologically closer to the genera *Neophyllobius* and *Tycherobius* (based on proximal solenidion on leg tarsi I–II) and distinct from *Acamerobia*, *Camerobia*, *Bisetolobius* and *Decaphyllobius* (based on the distal solenidion on leg tarsi I–II). It can be further distinguished from *Neophyllobius* and *Tycherobius* due to the presence of one midventral seta distally on leg tarsi I–II vs. two midventral setae on leg tarsi I–II in later two genera.

**Etymology:** The generic epithet is derived from the diagnostic character of one midventral seta on all leg tarsi (*mono* = one)

The new genus *Monobius* includes two species *M*. *meyerae* [16] and *M*. *electrus* [9]. Both species were originally described in the genus *Neophyllobius*.

#### 3.3.1. *Monobius meyerae* (Bolland) comb. nov.

*Neophyllobius meyerae* Bolland, 1991:63 [16].

**Remarks:** The species *M*. *meyerae* (Bolland) is referred to the new genus *Monobius* due to presence of one midventral seta on all leg tarsi. The species can be differentiated from the second species of the genus *M*. *electrus* (Żmudziński) based on number of setae on femur III (2 vs. 3), number of dorsal body setae (15 vs. 14) and state of ***pdx*** setae (present vs. absent).

**Distribution:** South Africa

#### 3.3.2. *Monobius electrus* (Żmudziński) comb. nov.

*Neophyllobius electrus* Żmudziński, 2020:3 [9].

**Remarks:** The species *M*. *electrus* (Żmudziński) is referred to the new genus *Monobius* due to presence of one midventral seta on all leg tarsi.

**Distribution:** Fossil preserved in Baltic Amber, Poland

### 3.4. Genus Tycherobius Bolland

*Tycherobius* Bolland, 1986: 205 [2].

*Tillandsobius* Bolland, 1986: 205; synonym nov.

**Diagnosis:** Two midventral setae on tarsi I–II, not present in a longitudinal line,

**Remarks:** The monospecific genus *Tillandsobius* was erected by Bolland [2] and it was distinguished from *Tycherobius* only by difference in number of setae on tibiae I–IV. As mentioned earlier, tibial setal counts are variable among the species of the genus *Tycherobius* and cannot be considered as a generic diagnostic character (Table 1).

#### *Tycherobius floridensis* (Bolland) comb. nov.

*Tillandsobius floridensis* Bolland, 1986:205 [2].

*Neophyllobius floridensis* McGregor, 1950:61 [10].

**Remarks:** The species *T*. *floridensis* resembles all 25 species of the genus based on two midventral setae on leg tarsi I–II not in a longitudinal line and leg tarsi III–IV, each, always with one midventral seta. It is closely related to the species *T*. *rhytis* based on tibiae I–II with 8–7 setae. However, it differs from later due to differences in number setae on tibiae III–IV (6–6 vs 7–7), tarsi I–IV (10–10–7–7 vs. 7–7–8–8), femur II–III (3–2 vs. 4–3) and differences in length of dorsal body setae (less than half the distance to the setae next in line vs. reaching to the base of setae next in line).

**Distribution:** Florida, USA

### 3.5. Genus Neophyllobius Berlese

*Neophyllobius* Berlese, 1886:19 [1].

**Type species:** *Neophyllobius elegans* Berlese, 1886:19 [1].

**Diagnosis:** Two midventral setae on tarsi I–II, present in a longitudinal line.

### 3.6. Subgenera in the Genus Neophyllobius

Among the species of the genus *Neophyllobius*, 114 species have two midventral setae on all leg tarsi. In contrast, 14 species have one midventral seta on leg IV and seven species with no such information available [6,8,9,16]. In the present study, the genus *Neophyllobius* is categorized in two new subgenera; *Neophyllobius* Berlese and *Monophyllobius* Mirza, based on the number of midventral setae on leg tarsi III–IV.

#### 3.6.1. New subgenus *Neophyllobius* Berlese

urn:lsid:zoobank.org:act:D34B5F98-1F09-45C8-8C64-62CA683C154E

**Type species:** *Neophyllobius elegans* Berlese, 1886:19 [1].

**Diagnosis:** Leg tarsi III–IV always with two midventral setae

**Number of species included:** 114

#### 3.6.2. New subgenus *Monophyllobius* Mirza

urn:lsid:zoobank.org:act:8648D2D3-7F38-442A-8C15-680FB8D560A7

**Type species:** *Neophyllobius texanus* McGregor, 1950:66 [10].

**Diagnosis:** Leg tarsus III often and tarsus IV always with one midventral seta

**Etymology:** The subgeneric epithet refers to the presence of one midventral seta on leg tarsi III and IV (*mono* = one)

**Number of species included:** 14

### 3.7. Redescriptions

The present research reported five new records of camerobiid mites from Saudi Arabia, viz; *N*. *combreticola*, *N*. *lorestanicus*, *N*. *denizliensis*, *T*. *emadi* and *C*. *southcotii*. The species *N*. *combreticola* along with two previously reported species, viz; *N*. *muscantribii* and *N*. *fissus* are redescribed in detail. In addition, the species *N*. *lorestanicus* and *N*. *denizliensis* were previously misidentified as *N*. *communis* and *N*. *hispanicus*, respectively.

#### 3.7.1. *Neophyllobius combreticola* Bolland

*Neophyllobius combreticola* Bolland, 1991:196 [16]; Beyzavi et al., 2013:393 [34].

Redescription (Figure 1, Figure 2, Figure 3, Figure 4, Figure 5, Figure 6, Figure 7, Figure 8 and Figure 9)

Female (*n* = 3)

**Figure 1:**
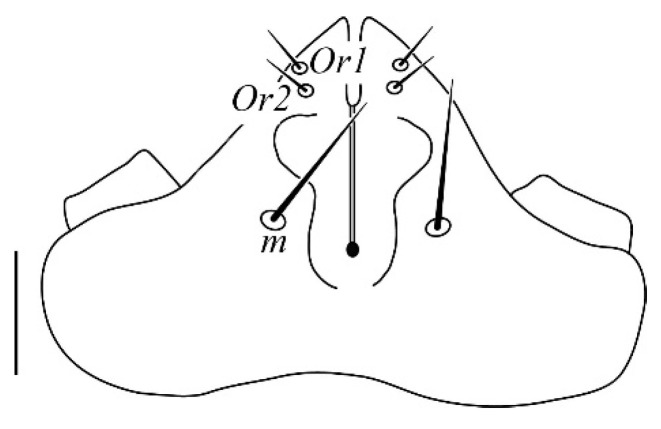
*Neophyllobius combreticola*. Female. Gnathosoma. Scale bar: 20 µm.

**Figure 2:**
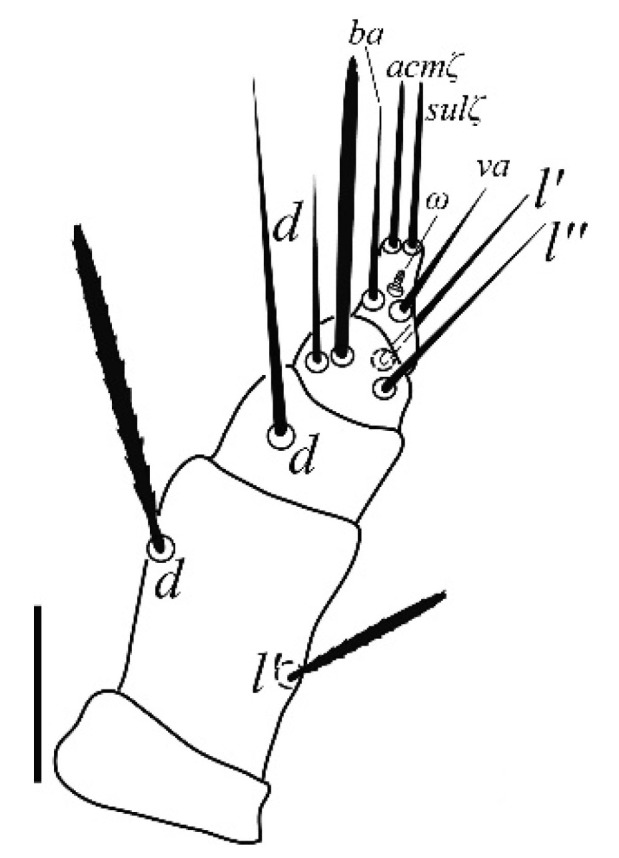
*Neophyllobius combreticola*. Female. Palp. Scale bar: 10 µm.

**Gnathosoma** (Figure 1 and Figure 2): 59–64 long, subcapitulum (Figure 1) with a subcapitular seta *m* 20–23 and two pairs of adoral setae *Or1* 7–9 and *Or2* 8–9, these three pairs simple and slender, chelicerae 24–28 long, palp five–segmented (Figure 2) with the following chaetotaxy: trochanter without setae; femora with two serrated setae, *d* 9–11 and *l’* 34–38, genua with one long, slender, simple dorsal seta *d* 35–38 (Figure 2), tibiae with three tactile setae (*l’*, *l”* and *d*) and one sword-like seta, tarsus with two eupathidia (*acmζ* and *sulζ*), two simple setae (*ba* and *va*) and one small solenidion (*ω*) (Figure 2).

**Figure 3:**
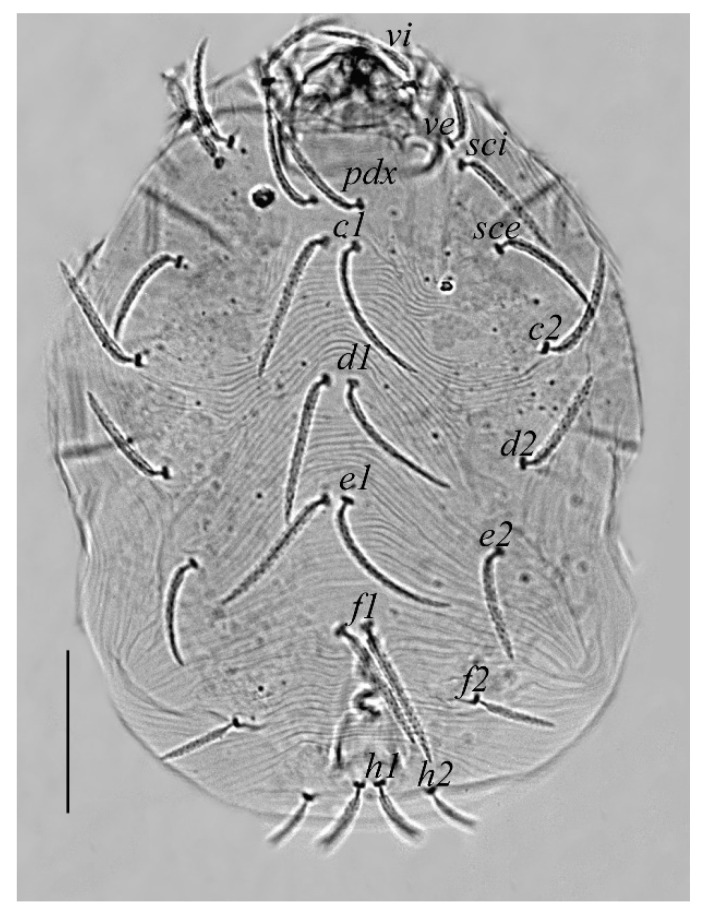
*Neophyllobius combreticola*. Female. Dorsum. Scale bar: 100 µm.

**Dorsum** (Figure 3): 369–378 long (excluding gnathosoma), integument transversely striated between all dorso–central setae, with 15 pairs of finely serrated setae set on small tubercles, all dorso–central setae longer than distance to the setae next in-line, two pairs of eyes positioned between setae *sci* and *sce*. Length of setae: *vi* 60–63, *ve* 65–71, *sci* 64–67, *sce* 66–70, *pdx* 75–82, *c1* 85–89, *c2* 64–71, *d1* 80–86, *d2* 60–65, *e1* 78–84, *e2* 59–65, *f1* 76–81, *f2* 45–49, *h1* 40–47, *h2* 34–38. Distances between setae: *pdx*–*pdx* 24–26, *c1*–*c1* 16–19, *d1*–*d1* 14–17, *e1*–*e1* 10–13, *f1*–*f1* 11–13, *h1*–*h1* 8–10, *pdx*–*c1* 20–24, *c1*–*d1* 63–67, *d1*–*e1* 54–61, *e1*–*f1* 50–55, *f1*–*h1* 70–73, *pdx*–*d1* 84–90, *c1*–*e1* 119–127, *d1*–*f1* 110–120, *e1*–*h1* 120–125.

**Figure 4:**
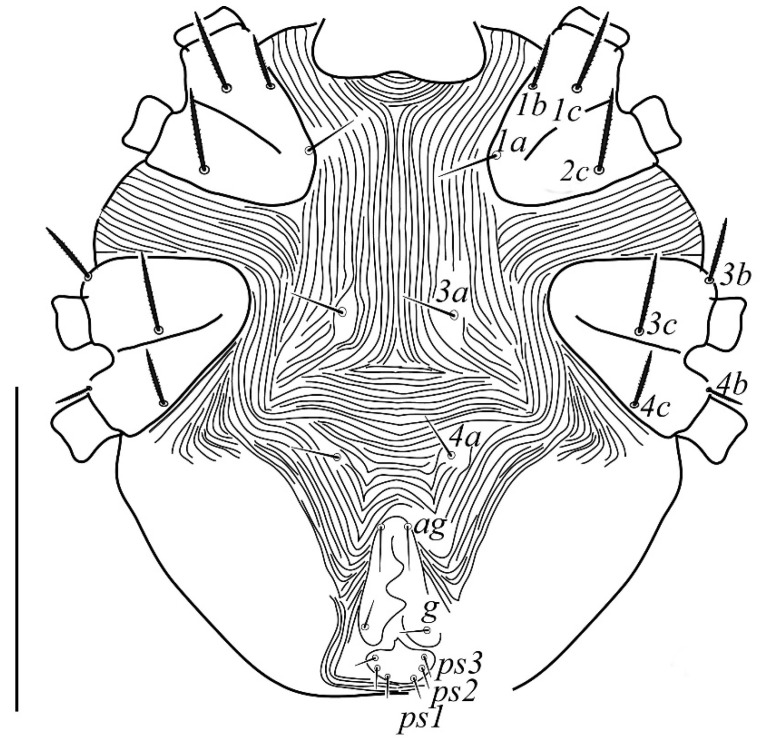
*Neophyllobius combreticola*. Female. Venter. Scale bar: 100 µm.

**Figure 5:**
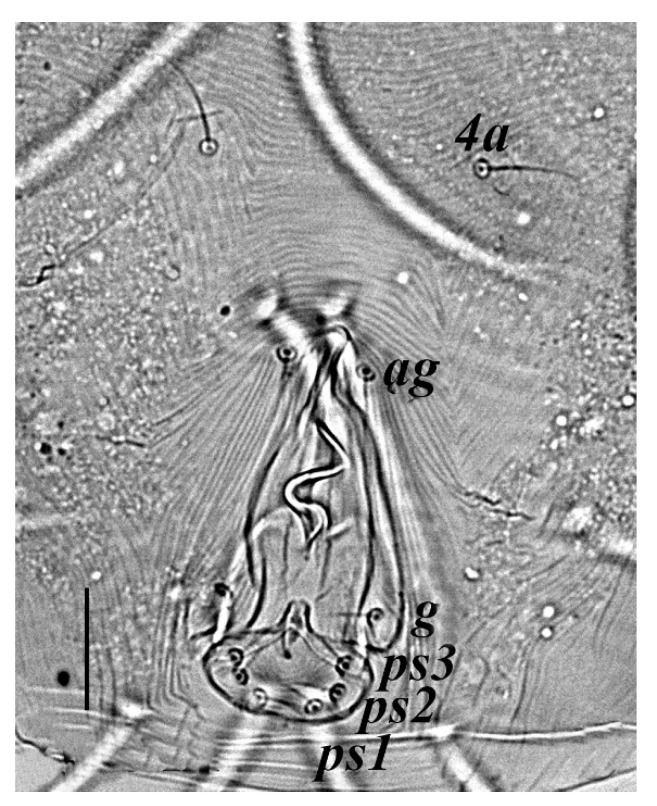
*Neophyllobius combreticola*. Female. Genital and anal region. Scale bar: 20 µm.

**Venter** (Figure 4 and Figure 5): Ventral idiosoma, striated longitudinally between coxae I–IV, coxal setae slender serrate, intercoxal setae *1a* present on the coxa I, coxa I grouped with coxa II and coxa III with IV but not completely fused (Figure 4). Length of setae: *1b* 22–26, *1c* 50–54, *2c* 42–45, *3b* 32–36, *3c* 42–44, *4b* 15–19, *4c* 23–25, intercoxal setae length: *1a* 26–28, *3a* 33–38, *4a* 15–17, one pair of aggenital setae (*ag*) present, genito-anal valves with a pair of genital setae (*g*) and three pairs of anal setae *ps1*, *ps2* and *ps3* (Figure 5).

**Figure 6:**
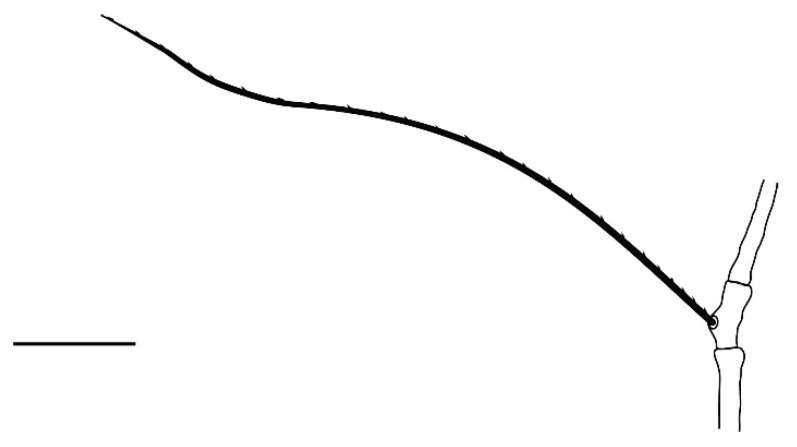
*Neophyllobius combreticola*. Female. Genu I. Scale bar: 50 µm.

**Figure 7:**
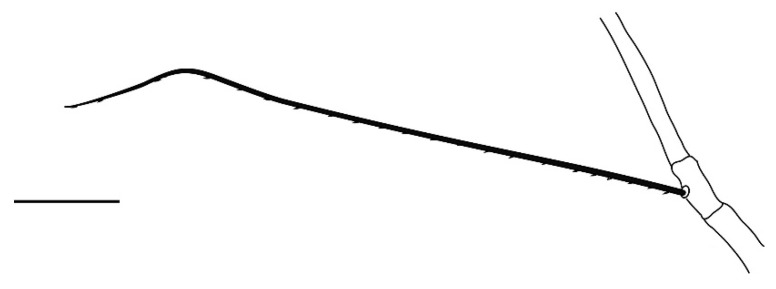
*Neophyllobius combreticola*. Female. Genu II. Scale bar: 50 µm.

**Figure 8:**
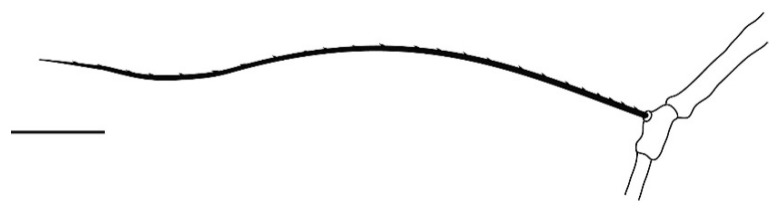
*Neophyllobius combreticola*. Female. Genu III. Scale bar: 50 µm.

**Figure 9:**
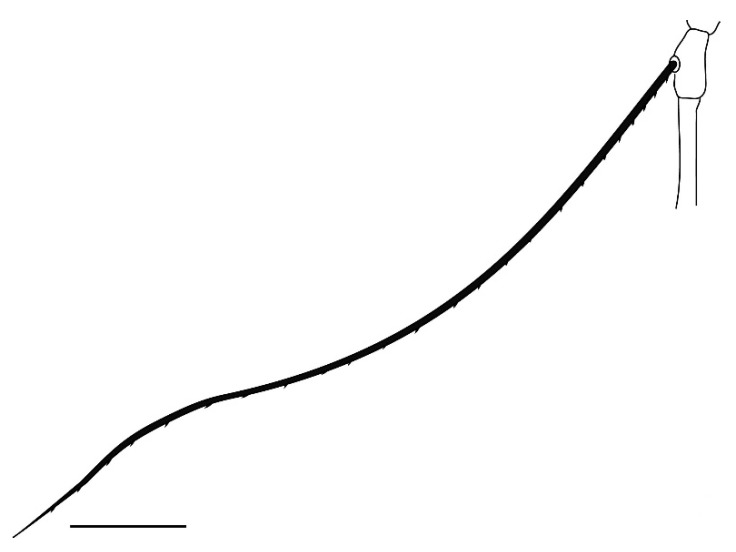
*Neophyllobius combreticola*. Female. Genu IV. Scale bar: 50 µm.

**Legs** (Figure 6, Figure 7, Figure 8 and Figure 9): Slender and long, lengths (excluding coxae and including ambulacra): leg I 568–572, leg II 500–510, leg III 510–518, leg IV 570–576. Leg segment chaetotaxy (solenidia in parenthesis) as follows: coxae 3–1–2–2, leg segment chaetotaxy (solenidia in parenthesis) as follows: coxae: 3–1–2–2, trochanters 1–1–1–1, femur 4–3–2–2, genu 1(*κ*)–1(*κ*)–1–1, tibiae 9(*φ*)–8(*φ*)–8(*φ*)–7(*φ*), tarsi 10(*ω*)–10(*ω*)–8–8. All tarsi with ambulacrum bearing a pair of claws and an empodium with two rows of tenent hairs, all genu setae long and whip like, dorsal seta on genu I 290–300 (Figure 6), genu II 300–305 (Figure 7), genu III 336–342 (Figure 8), genu IV 358–360 (Figure 7). All leg tarsi with two in-line midventral setae and tarsi I–II with a basal solenidion.

**Remarks:** The species *Neophyllobius combreticola* is a new species record for the camerobiid mite fauna of Saudi Arabia. It belongs to the *Neophyllobius* subgenus nov. based on presence of two in-line midventral setae on tarsi I–IV. The specimens are almost similar to the original description [16] except all dorsal body setae are longer (5–10 µm) in the current collection. Furthermore, Alatawi and Kamran [27] reported *N*. *hispanicus*, which was a misidentification of this redescribed species.

**Material Examined:** Two females, unidentified plant, Al–Bashyer, Asir, SA, 19°15.884′ N, 42°05.261′ E, 29 October 2019, coll. M. Kamran and H. M. S. Mushtaq; one female, Acacia spp., Al–Baha, SA, 20°12.653′ N, 41°37.970′ E, 27 October 2019, coll. M. Kamran and H. M. S. Mushtaq.

**Previous Distribution:** Iran, South Africa

#### 3.7.2. *Neophyllobius fissus* de Leon

*Neophyllobius fissus* de Leon, 1967:31 [35]; Bolland, 1991:212 [16].

Redescription (Figure 10, Figure 11, Figure 12, Figure 13, Figure 14, Figure 15, Figure 16 and Figure 17)

Female (*n* = 3)

**Figure 10:**
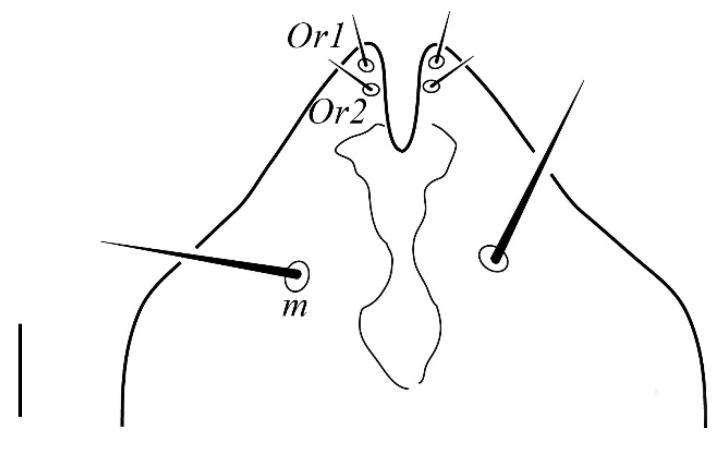
*Neophyllobius fissus*. Female. Gnathosoma. Scale bar: 20 µm.

**Gnathosoma** (Figure 10): 51–55 long, subcapitulum with a subcapitular seta *m* 24–25 long and two pairs of adoral setae *Or1* 7 and *Or2* 8, these three pairs simple and slender, chelicerae 21 long, palp five-segmented with the following chaetotaxy: trochanter without setae; femora with two serrated setae, *d* 22–23 and *l′* 31–34, genua with one long, slender, simple dorsal seta *d* 34–35, tibiae with three tactile setae (*l′*, *l”* and *d*) and one sword–like seta, tarsus with two eupathidia (*acmζ* and *sulζ*), two simple setae (*ba* and *va*) and one small solenidion (*ω*).

**Figure 11:**
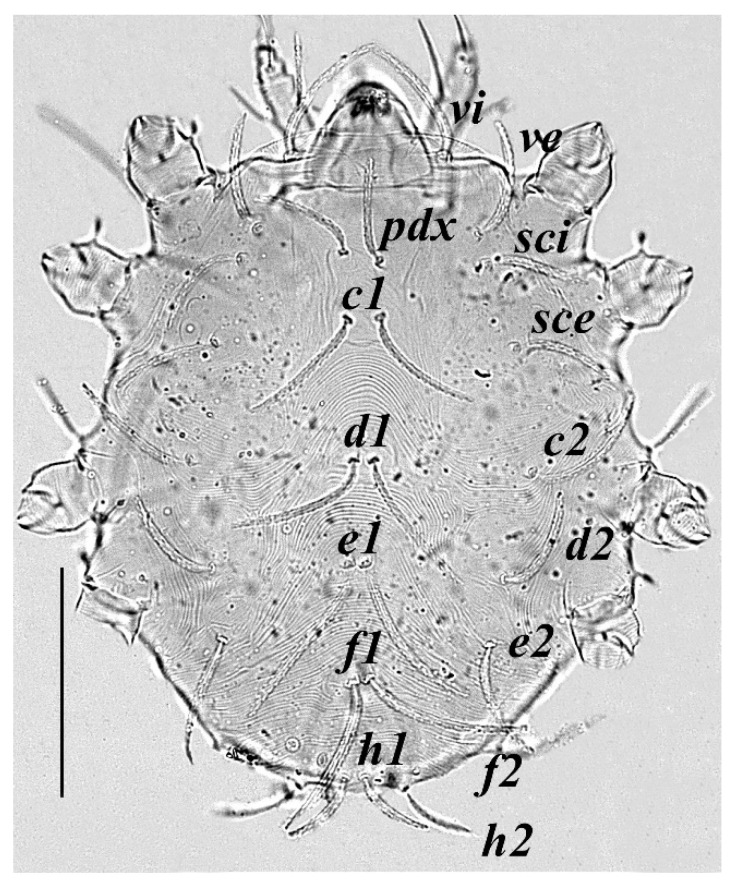
*Neophyllobius fissus*. Female. Dorsum. Scale bar: 100 µm.

**Dorsum** (Figure 11): 260–271 long (excluding gnathosoma), integument transversely striated between all dorso-central setae, with 15 pairs of finely serrated setae set on small tubercles, all dorso-central setae longer than distance to the setae next in-line, two pairs of eyes positioned between setae *sci* and *sce*. Length of setae: *vi* 49–51, *ve* 46–48, *sci* 40–44, *sce* 36–40, *pdx* 40–45, *c1* 52–54, *c2* 15–19, *d1* 59–63, *d2* 41–45, *e1* 78–84, *e2* 46–48, *f1* 63–67, *f2* 45–47, *h1* 28–32, *h2* 35–39. Distances between setae: *pdx*–*pdx* 14–16, *c1*–*c1* 11–13, *d1*–*d1* 9–10, *e1*–*e1* 6–7, *f1*–*f1* 6–8, *h1*–*h1* 8–10, *pdx*–*c1* 20–22, *c1*–*d1* 55–57, *d1*–*e1* 40–44, *e1*–*f1* 44–48, *f1*–*h1* 38–41, *pdx*–*d1* 78–83, *c1*–*e1* 99–103, *d1*–*f1* 85–90, *e1*–*h1* 83–85.

**Figure 12:**
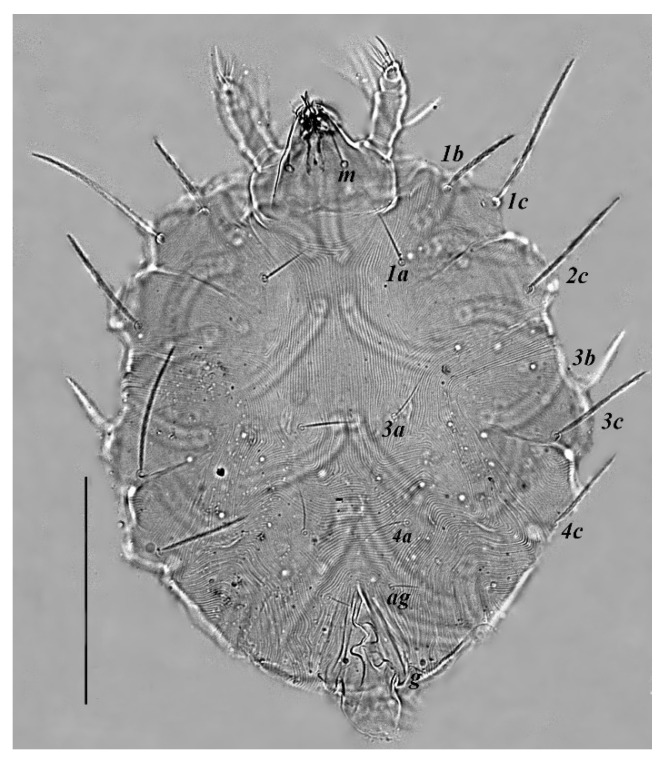
*Neophyllobius fissus*. Female. Venter. Scale bar: 100 µm.

**Figure 13:**
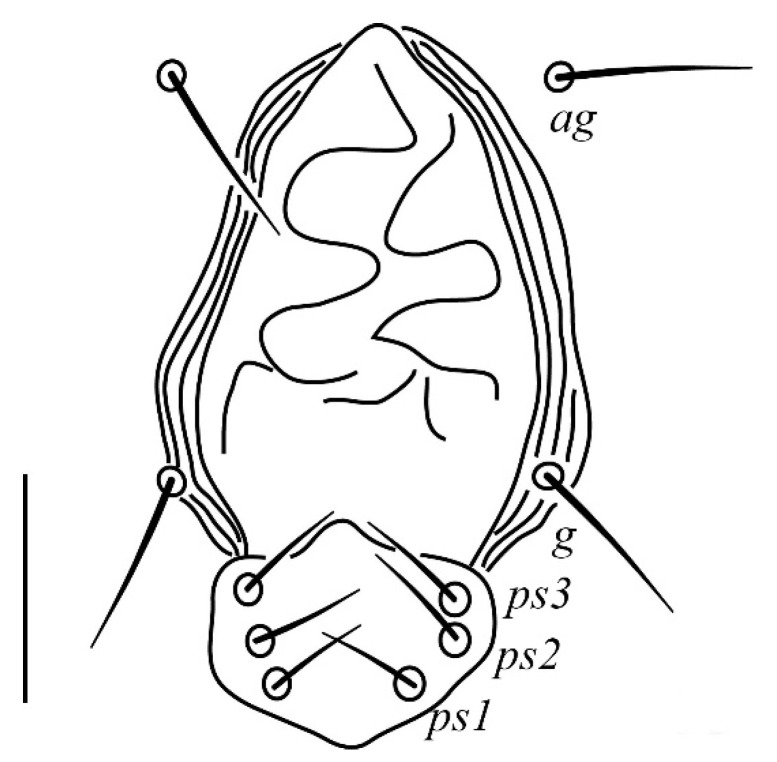
*Neophyllobius fissus*. Female. Genital and anal region. Scale bar: 20 µm.

**Venter** (Figure 12 and Figure 13): Ventral idiosoma, striated longitudinally between coxae I–IV, coxal setae slender serrate, intercoxal setae *1a* present on the coxa I, coxa I grouped with coxa II and coxa III with IV but not completely fused. Length of setae: *1b* 40–43, *1c* 73–75, *2c* 57–62, *3b* 30–33, *3c* 63–67, *4b* 10–11 (curved dorsally), *4c* 43–44, intercoxal setae length: *1a* 32–35, *3a* 34–46, *4a* 25–27 (Figure 12), one pair of aggenital setae (*ag*) 14–15, genito-anal valves with a pair of genital setae (*g*) 10–13 and three pairs of anal setae *ps1*, *ps2* and *ps3* (Figure 13).

**Figure 14:**
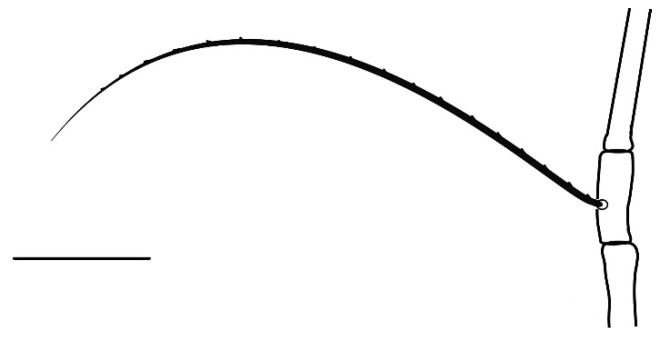
*Neophyllobius fissus*. Female. Genu I. Scale bar: 50 µm.

**Figure 15:**
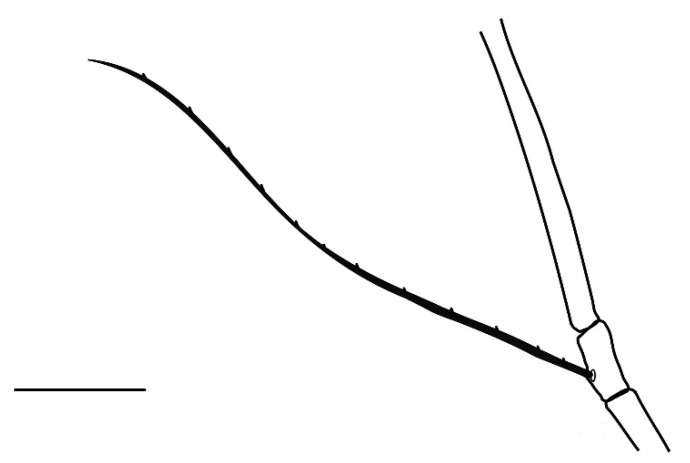
*Neophyllobius fissus*. Female. Genu II. Scale bar: 50 µm.

**Figure 16:**
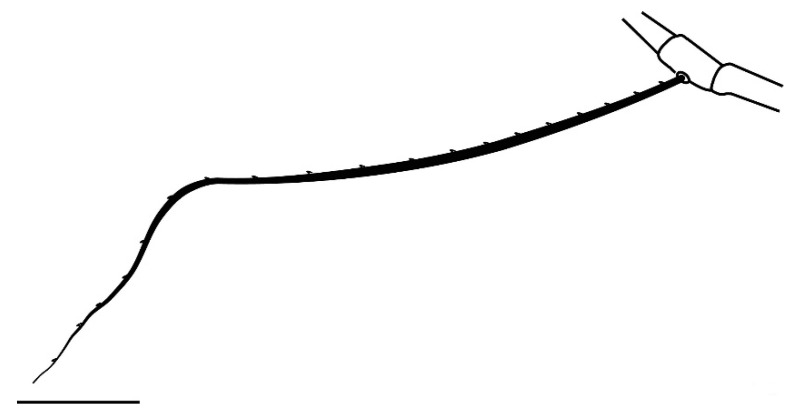
*Neophyllobius fissus*. Female. Genu III. Scale bar: 50 µm.

**Figure 17:**
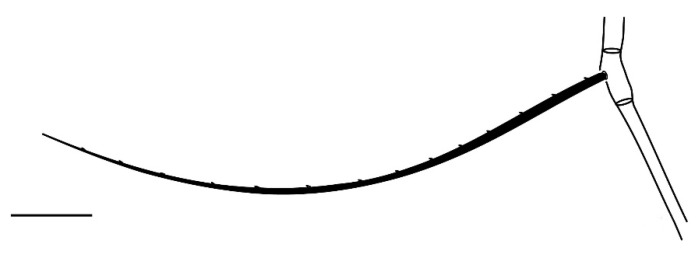
*Neophyllobius fissus*. Female. Genu IV. Scale bar: 50 µm.

**Leg** (Figure 14, Figure 15, Figure 16 and Figure 17): Slender and long, lengths (excluding coxae and including ambulacra): leg I 536–540, leg II 450–460, leg III 485–493, leg IV 529–534. Leg segment chaetotaxy (solenidia in parenthesis) as follows: coxae 3–1–2–2, leg segment chaetotaxy (solenidia in parenthesis) as follows: coxae: 3–1–2–2, trochanters 1–1–1–1, femur 4–3–2–2, genu 1(*κ*)–1(*κ*)–1–1, tibiae 9(*φ*)–8(*φ*)–8(*φ*)–7(*φ*), tarsi 10(*ω*)–10(*ω*)–8–8. All tarsi with ambulacrum bearing a pair of claws and an empodium with two rows of tenent hairs, all genu setae long and whiplike, dorsal seta on genu I 218–220 (Figure 14), genu II 233–237 (Figure 15), genu III 313–318 (Figure 16), genu IV 377–382 (Figure 17). All leg tarsi with two in-line midventral setae and tarsi I–II with a basal solenidion.

**Remarks:** The species *Neophyllobius fissus* was previously reported from Saudi Arabia [27]. It belongs to the *Neophyllobius* subgenus nov. based on the presence of two in-line midventral setae on tarsi I–IV. The specimens are almost similar to what was described in the original description [16] except some variations in the length of dorsal body setae.

**Material Examined:** Two females, date palm (Arecaceae), Al–Qatif, SA, 26°34.36.7**′** N, 49°59.07.1**′** E, 25 January 2014 and 11 December 2013, coll. Kamal Alsahwan; one female, date palm (Arecaceae), Al–Imam University, Riyadh, SA, 24°48.785**′** N, 46°42.142**′** E, 19 March 2011, coll. Kamal Alsahwan.

**Previous Distribution:** Trinidad

#### 3.7.3. *Neophyllobius muscantribii* Bolland

*Neophyllobius muscantribii* Bolland, 1991:73 [16].

*Neophyllobius eragrostidis* Bolland, 1991:73 [16]; *Eragrostis* (plant), syn. by du Toit, Theron and Ueckermann, 1998 [5].

Redescription (Figure 18, Figure 19, Figure 20, Figure 21, Figure 22, Figure 23, Figure 24 and Figure 25)

Female (*n* = 3)

**Figure 18:**
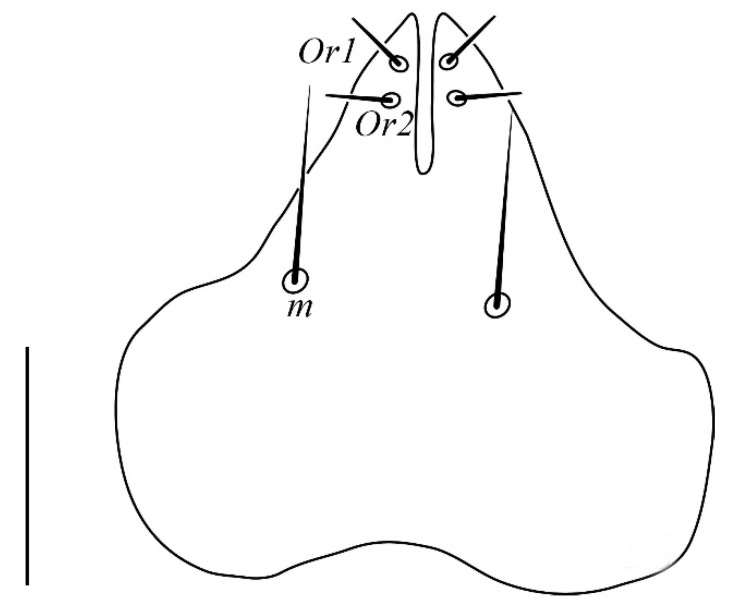
*Neophyllobius muscantribii*. Female. Gnathosoma. Scale bar: 20 µm.

**Gnathosoma** (Figure 18): 54–60 long, subcapitulum with a subcapitular seta *m* 12–17 and two pairs of adoral setae *Or1* 6–7 and *Or2* 7–8, these three pairs simple and slender, chelicerae 14–16 long, palp five-segmented with the following chaetotaxy: trochanter without setae; femora with two serrated setae, *d* 8–11 and *l’* 23–25, genua with one long, slender, simple dorsal seta *d* 20–22, tibiae with three tactile setae (*l’*, *l”* and *d*) and one sword-like seta, tarsus with two eupathidia (*acmζ* and *sulζ*), two simple setae (*ba* and *va*) and one small solenidion (*ω*).

**Figure 19:**
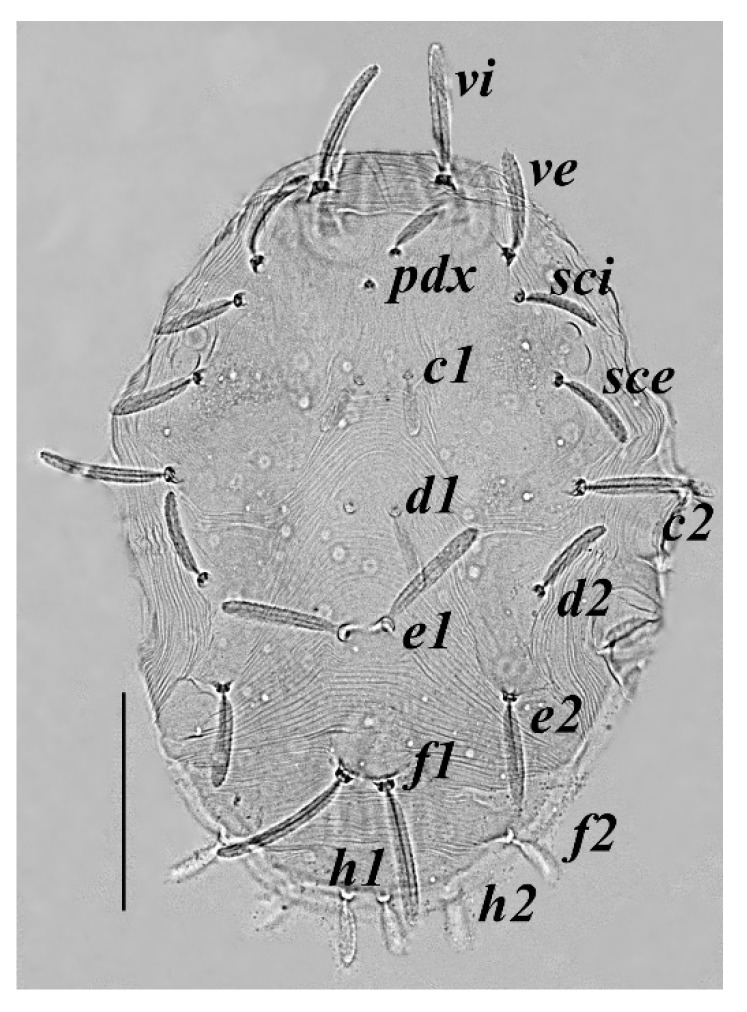
*Neophyllobius muscantribii*. Female. Dorsum. Scale bar: 100 µm.

**Dorsum** (Figure 19): 355–361 long (excluding gnathosoma), integument transversely striated between all dorso-central setae, with 15 pairs of finely serrated setae, broadly lanceolate and set on small tubercles, almost all dorso-central setae shorter than distance to the setae next in-line, two pairs of eyes positioned between setae *sci* and *sce*. Length of setae: *vi* 50–53, *ve* 35–38, *sci* 33–37, *sce* 37–41, *pdx* 30–35, *c1* 28–30, *c2* 56–58, *d1* 33–37, *d2* 36–41, *e1* 50–56, *e2* 35–40, *f1* 55–57, *f2* 32–34, *h1* 27–28, *h2* 24–27. Distances between setae: *pdx*–*pdx* 15–18, *c1*–*c1* 20–21, *d1*–*d1* 18–20, *e1*–*e1* 15–20, *f1*–*f1* 12–16, *h1*–*h1* 14–15, *pdx*–*c1* 43–48, *c1*–*d1* 53–58, *d1*–*e1* 58–60, *e1*–*f1* 47–50, *f1*–*h1* 56–58, *pdx*–*d1* 100–105, *c1*–*e1* 118–119, *d1*–*f1* 110–111, *e1*–*h1* 100–108.

**Figure 20:**
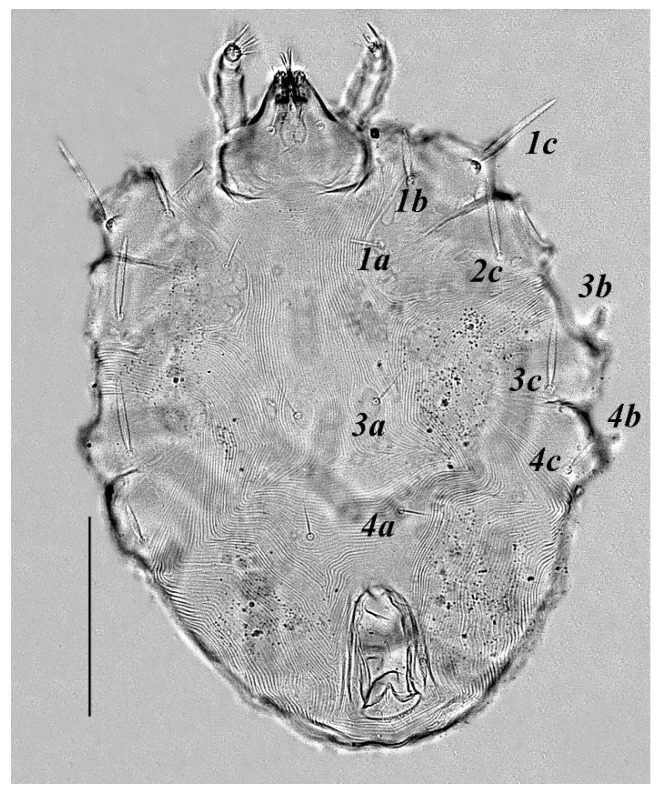
*Neophyllobius muscantribii*. Female. Venter. Scale bar: 100 µm.

**Figure 21:**
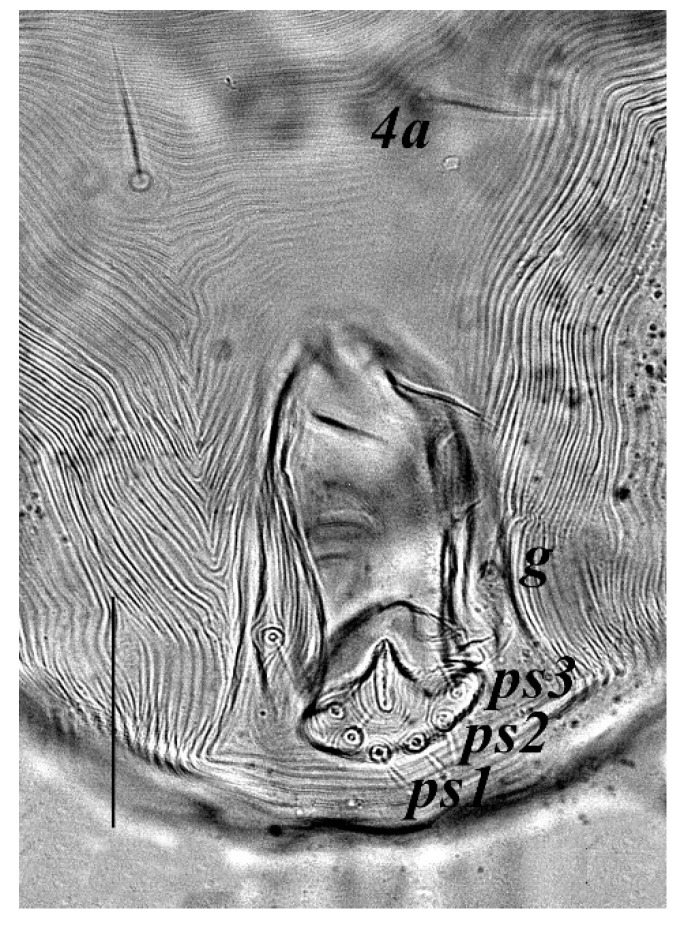
*Neophyllobius muscantribii*. Female. Genital and anal region. Scale bar: 20 µm.

**Venter** (Figure 20 and Figure 21): Ventral idiosoma, striated longitudinally between coxae I–IV, coxal setae slender serrate, intercoxal setae *1a* present on the coxa I, simple, hair like (Figure 20). Length of setae: *1b* 23–24, *1c* 50–53, *2c* 38–40, *3b* 15–17, *3c* 40–42, *4b* 10–12, *4c* 21–22, intercoxal setae length: *1a* 15–17, *3a* 25–27, *4a* 12–14, one pair of aggenital setae (*ag*) 14–15 present, genito–anal valves with a pair of genital setae (*g*) 7–9 and three pairs of anal setae (*ps1*, *ps2* and *ps3*) (Figure 21).

**Figure 22:**
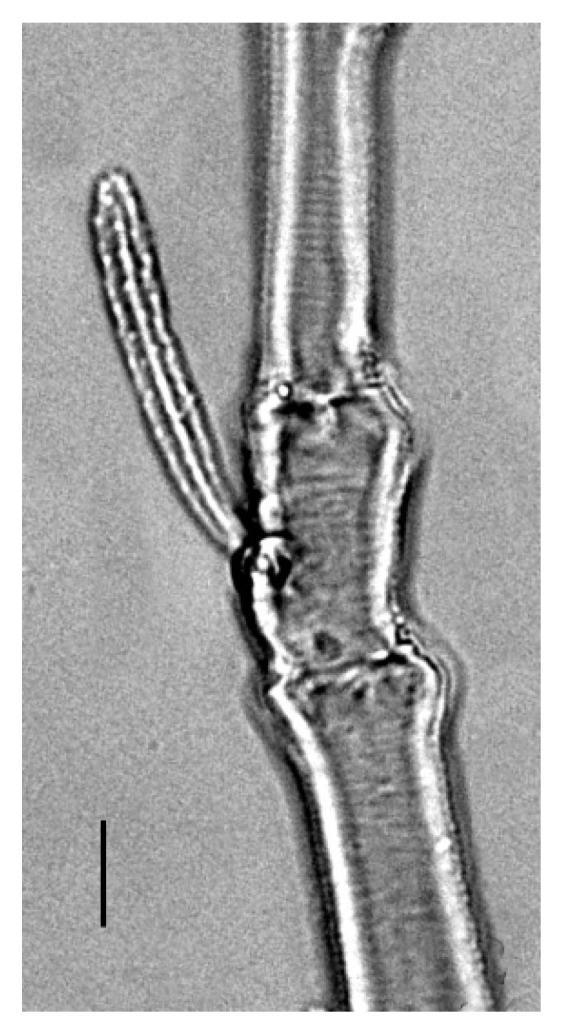
*Neophyllobius muscantribii*. Female. Genu I. Scale bar: 50 µm.

**Figure 23:**
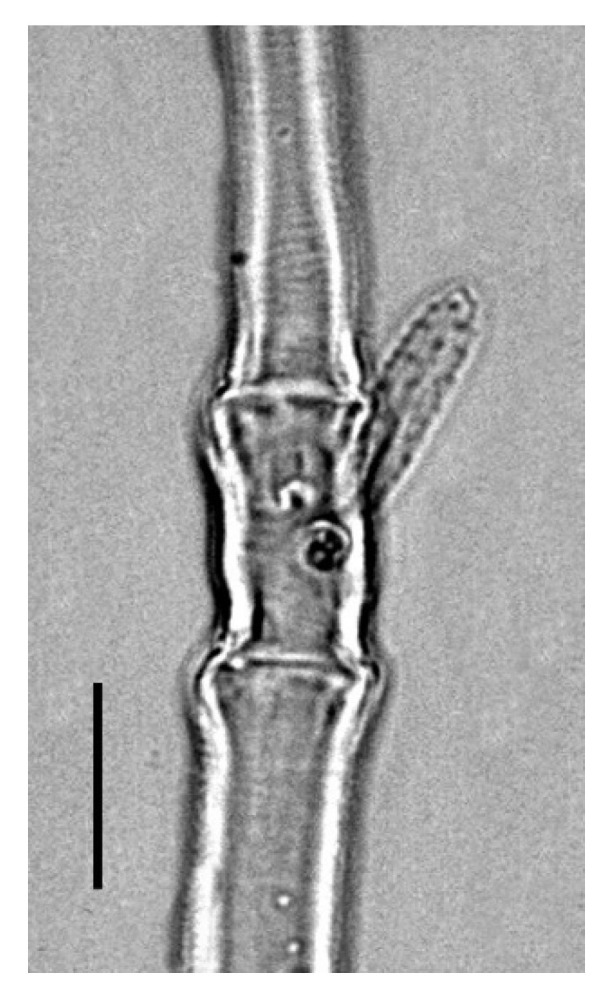
*Neophyllobius muscantribii*. Female. Genu II. Scale bar: 50 µm.

**Figure 24:**
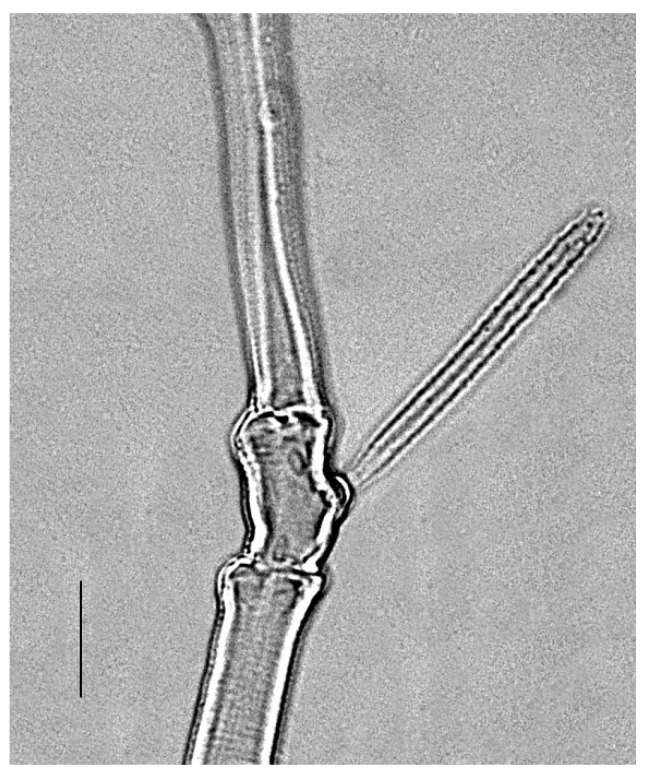
*Neophyllobius muscantribii*. Female. Genu III. Scale bar: 50 µm.

**Figure 25:**
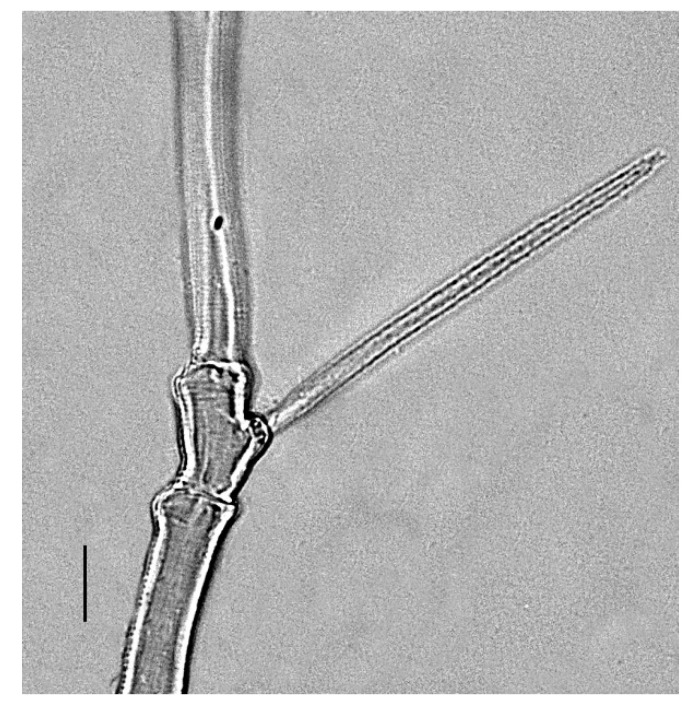
*Neophyllobius muscantribii*. Female. Genu IV. Scale bar: 50 µm.

**Leg** (Figure 22, Figure 23, Figure 24 and Figure 25): Slender and long, lengths (excluding coxae and including ambulacra): leg I 450–452, leg II 388–390, leg III 410–415, leg IV 460–466. Leg segment chaetotaxy (solenidia in parenthesis) as follows: coxae 3–1–2–2, leg segment chaetotaxy (solenidia in parenthesis) as follows: coxae: 3–1–2–2, trochanters 1–1–1–1, femur 4–3–2–2, genu 1(*κ*)–1(*κ*)–1–1, tibiae 9(*φ*)–8(*φ*)–8(*φ*)–7(*φ*), tarsi 10(*ω*)–10(*ω*)–8–8. All tarsi with ambulacrum bearing a pair of claws and an empodium with two rows of tenent hairs, dorsal seta on genu I (38–41) twice as long as the segment (Figure 22), seta on genu II (26–28) equal to the segment length (Figure 23), genu III seta (63–65) long, not reaching the first row of setae on tibia III (Figure 24), genu IV seta (118–120) also, not reaching the first row of setae on tibia IV (Figure 25). All leg tarsi with two in-line midventral setae and tarsi I–II with a basal solenidion.

**Remarks:** The species *Neophyllobius muscantribii* was previously reported from Saudi Arabia [27]. It belongs to the *Neophyllobius* subgenus nov. based on presence of two in-line midventral setae on tarsi I–IV. The specimens are almost similar to the brief original description [16].

**Material Examined:** 1 female, Grasses (Poaceae), Asir, SA, 19°15.884′ N, 42°05.261′ E, 29 October 2019, coll. M. Kamran and H. M. S. Mushtaq, 2 females, Kavi plant and Sider like, Al–Baha, SA, 19°59.807′ N, 41°25.715′ E, 25 April 2013, coll. Kamal Alsahwan.

**Previous Distribution:** South Africa

### 3.8. New Records

#### 3.8.1. *Neophyllobius lorestanicus* Khanjani, Hoseini, Yazdanpanah and Masoudian

*Neophyllobius lorestanicus* Khanjani, Hoseini, Yazdanpanah and Masoudian, 2014: 441 [36].

**Remarks:** The species *N*. *lorestanicus* is a new record for the camerobiid mite fauna of Saudi Arabia and no distinct morphological differences were found between Saudi Arabian specimens of *N*. *lorestanicus* and the original description. It belongs to the *Neophyllobius* subgenus nov. based on presence of two in-line midventral setae on tarsi I–IV.

**Material Examined:** Two females, date palm (Arecaceae), Irqa, Riyadh, SA, 24°41.130′ N, 46°34.550′ E, 11 December 2013, coll. Kamal Alsahwan; one female, Acacia spp, (Fabaceae). Al–Ahsa, SA, 25°55.371′ N, 48°59.037′ E, 22 October 2020, coll. J. H. Mirza, N. A. Elgoni, H. M. Sajid and M. W. Khan.

**Previous distribution**: Iran

#### 3.8.2. *Neophyllobius denizliensis* Akyol

*Neophyllobius denizliensis* Akyol, 2020:88 [8].

**Remarks:** The species *N*. *denizliensis* is a new record for the camerobiid mite fauna of Saudi Arabia. It was previously misidentified as *N*. *hispanicus* [27]. It belongs to the *Neophyllobius* subgenus nov. based on presence of two in-line midventral setae on tarsi I–IV. No morphological differences were found between Saudi Arabian specimens and original description of the *N*. *denizliensis*.

**Material Examined:** One female, *Tamarix* spp., (Tamaricaceae), Tabuk, SA, 28°30.653′ N, 36°28.168′ E, September 29, 2020, coll. J. H. Mirza, H. M. S. Mushtaq and E. M. Khan; one female, *Phoenix dactylifera* (Arecaceae), Irqa, Riyadh, SA, 13 December 2020, coll. Kamal Alsahwan; one female, grasses (Poaceae). Al–Imam University, Riyadh, SA, 24°48.785′ N, 46°42.142′ E, 7 April 2016, coll. M. Kamran.

**Previous distribution:** Turkey

#### 3.8.3. *Genus Tycherobius Bolland*

*Tycherobius* Bolland, 1986:205 [16].

**Type species**: *Neophyllobius lombardinii* Summers and Schlinger, 1955 [12].

***Tycherobius*** ***emadi* Khanjani, Hajizadeh, Ostovan and Asali Fayaz**

*Tycherobius emadi* Khanjani, Hajizadeh, Ostovan and Asali Fayaz, 2013:134 [37]; Hoseini and Khanjani, 2013:212 [38].

**Remarks:** The species *Tycherobius emadi* is first time reported from Saudi Arabia and is morphologically similar to the original description [37].

**Material Examined:** One female, soil, and leaf debris, Tabuk, SA, 26°58.271′ N, 49°40.220′ E, 19 October 2019, coll. M. Kamran and H. M. S. Mushtaq.

**Previous distribution:** Iran

#### 3.8.4. Genus *Camerobia* Southcott

*Camerobia* Southcott, 1957: 306 [4].

**Type species**: *Camerobia australis* Southcott, 1957 [4]

*Camerobia southcotti* Gerson

*Camerobia southcotti* Gerson, 1972: 502 [14].

**Remarks:** The species *Camerobia southcotti* is a new record for the camerobiid mite fauna in Saudi Arabia and is morphologically similar to the original description [14].

**Material Examined:** Three females, unidentified wild host plant, Jeddah, SA, 26°58.271′ N, 49°40.220′ E, 25 April 2016, coll. M. Kamran and J. H. Mirza.

**Previous distribution:** Israel

### 3.9. Additional Notes

The following 10 species are not included in the key either due to lack of available literature or incomplete descriptions/illustrations. The available but scarce information from different literatures about these species are summarized in the Table 2.

***Neophyllobius elegans*** **Berlese**

*Neophyllobius elegans* Berlese, 1886:19 [1]; 1900:288 [39]; Canestrini, 1889:457 [40]; Bolland, 1991:212 [16].

Bolland [16] reported that the type specimen was not available for study. Most of the characters provided by Bolland [16] were based on research papers of Berlese and Canestrini and those are also provided in the Table 2

***Neophyllobius guajavae*** **Chatterjee and Gupta**

*Neophyllobius guajavae* Chatterjee and Gupta, in Gupta, 2002:38 [41].

The description and illustrations provided are very poor and didn’t help to place the species in the diagnostic key provided in present work. Although, the original authors have compared the species with *N*. *natalensis*.

Based on the present work, the species *N*. *guajavae* belongs to the new subgenus *Monophyllobius* based on tarsi III–IV, each, with one midventral seta (as only illustrated) and closely resembling *N*. *variegata*. The dorso-central setae in both the species are short where *c1* is 1/2 and 1/3 the length of *c1*–*d1* in *N*. *guajavae* and *N*. *variegata*, respectively.

***Neophyllobius hyderabadensis*** **Indra, Rao and Thakur**

*Neophyllobius hyderabadensis* Indra, Rao and Thakur, 1980:121 [42].

The original published description and illustration were not found. The first author contacted Mr. Mahran Zeity who published a new camerobiid species from India, but was not able to get any information about *N*. *hyderabadensis* (personal communication). Hence, it was not possible to provide any conclusive remarks on this species.

***Neophyllobius mexicanus*** **McGregor**

*Neophyllobius mexicanus* McGregor, 1950:49 [10]; de Leon, 1958:181 [35]; Bolland, 1991:218 [16].

The original description and illustration provided by McGregor [10] were not enough to compare with other species of the genus. de Leon [35] and Bolland [16] were unable to retrieve the type specimen hence they did not provide any details on species description. McGregor [10] and de Leon [35] mentioned that the dorso-central setae are shorter than the distance between the setae next in line and that genual setae are longer than twice the length of respective genua.

***Neophyllobius ornatus*** **Womersley**

*Neophyllobius ornatus* Womersley, 1940:248 [43]; Bolland, 1991:219 [16]; Fan and Walter, 2011:7 [3].

The status of this species is unresolved. The type specimen is lost as reported by Bolland [16] and Fan and Walter [3]. The original description and illustration provided by Womersley [43] were poor and both the previous references have tried to guess the leg chaetotaxy. Fan and Walter [3] mentioned two midventral setae on all leg tarsi. Bolland [16] suggested that this species was close to *N*. *fissus*, *N*. *aegyptium*, *N*. *niloticus* due to the femur IV with one seta.

Based on available information and classification of species in the present work, *N*. *ornatus* could belong to group of eight species (*N*. *fissus*, *N*. *aegyptium*, *N*. *niloticus*, *N*. *lalbaghensis*, *N*. *womersleyi*, *N*. *bamiensis*, *N*. *punctulatus*, *N*. *ferrugineus*) where all leg tarsi have two mid-ventral setae and femur I–IV with 4–3–2–1 setae.

***Neophyllobius saxatilis*** **Halbert**

*Neophyllobius saxatilis* Halbert, 1923:384 [44]; van Eyndhoven, 1938:25 [45]; Bolland, 1991:214 [16].

van Eyndhoven [45] reported this species with few morphological characters and illustrations. Bolland [16] also provided short description and illustration from a co-type specimen as the type slide was in bad condition.

In the present study, the *N*. *saxatlilis* could be placed among 48 species of the new subgenus *Neophyllobius* having all leg tarsi with two in-line midventral setae, femur I–IV with 4–3–2–2 setae, palp tarsus having two setae and two eupathidia, one solenidion on leg tarsi I–IV, dorsal setae ***c1*** reaching the bases of setae ***d1***, setae ***d1*** not reaching the bases of setae ***f1***.

***Neophyllobius summersi*** **McGregor**

*Neophyllobius summersi* McGregor, 1950:67 [10]; Bolland, 1991:218 [16].

McGregor [10] first time described this species with a unique character of palpfemur with three setae. All other *Neophyllobius* species, to date, do not contain such palp chaetotaxy. Bolland [16] added some other characters and confirmed this unique morphological feature. Although, Zaher and Gomaa [46] considered *N*. *mangiferus* to be close to *N*. *summersi* and differed from the latter on the basis of length of dorso-central setae and genu I–IV setae. However, it was not possible to include *N*. *summersi*, in the present diagnostic key, even with its unique character of palpfemur.

***Neophyllobius vanderwieli*** **Oudemans**

*Neophyllobius vanderwieli* Oudemans, 1926:121 [47]; Bolland, 1991:219 [16].

Bolland [16] mentioned, description based on a male specimen, that there are two solenidion distally on tibia I. We could consider this species close to three other species (*N*. *kamalii*, *N*. *karabagiensis* and *N*. *sycomorus*) which share the same character.

***Neophyllobius sp.***

This unidentified species was reported from Yemen by Ueckermann et al. [48]. The authors provided no information (morphological description or illustrations) except the material examined.

***Neophyllobius sp***.

This unidentified species was mentioned by Ripka et al. [49], which was collected during a survey of Hungarian mite fauna. The authors suspected this species was near to *N*. *dichantii*, but no morphological data was provided

### 3.10. Ventral idiosoma chaetotaxy

Bolland [2,16] provided a detailed family description and comprehensively reviewed the genus *Neophyllobius*, with 50 new species and 35 species redescriptions. A detailed genus diagnosis was given including; the ventral idiosoma with three pairs of ventral setae, two pairs of genital setae and three pairs of anal setae. In the remarks, the author stated that pregenital setae were never found in any other species of *Neophyllobius*, described until that time, in contrast to what was illustrated by Kuznetsov and Livshits [33]; presence of a paired pregenital setae.

It is difficult to discern the ventral idiosomal chaetotaxy from the genus diagnosis provided by Bolland [16] as that huge taxonomic review of the genus lacks ventral morphology of all 85 described and illustrated species. The presence of three pairs of ventral setae, as stated by the author, is confusing because the setae *1a* is found on the coxa I in all species of *Neophyllobius* and it was counted along with coxal setal count. The similar concept is evident in the genus diagnosis provided by Bolland [16] and all the species of *Neophyllobius* described to date. If the three pairs of ventral setae include the coxal seta *1a*, then it supports the absence of aggenital seta (*ag*) in adult females as reported by Bolland [16]. However, if the three pairs of ventral setae do not include coxal seta *1a*, then the total number of setae on the ventral idiosoma according to Bolland [16], add up to five pairs (paired three ventral and two genital setae). This has not been reported in any species of the seven camerobiid genera.

Adding to this confusion, four different ventral idiosoma descriptions and illustrations represented by 55 *Neophyllobius*, one each of *Tycherobius* (*T*. *dazkiriensis*), *Bisetolobius* (*B*. *varius*) species are available till date. For the ease of understanding, we here consider them as four different ventral idiosoma chaetotaxy (VIC) descriptions. All these cases have a total of four pairs of setae excluding coxal and anal setae. The species representing these cases are also presented in the Table 3. The VIC #1 is represented by 16 species where paired *3a*, *4a*, *ag* and *g* setae are present. The VIC #2 is represented by 10 species where paired *3a* is absent. The VIC #3 is represented by 14 species of *Neophyllobius* and a species, *T*. *dazkiriensis*, where paired setae *4a* is absent. The VIC #4 includes 13 species of *Neophyllobius* and a species *B*. *varius*, where paired aggenital setae (*ag*) is absent. It is noteworthy that, in the cases 2–4, where either one of the paired setae was absent, two pairs of genital setae were described and illustrated (Table 3). In addition, the discrepancies were also found between the description and illustration of four species for *3a*–*4a*–*ag*–*g* setae including *N*. *bamiensis* (0–1–1–2 vs. 1–1–1–1, respectively), *N*. *lorioi* (genitalia with two setae vs. no illustration, respectively), *N*. *saberi* (0–0–2–2 vs. 0–1–1–2, respectively) and *N*. *zolfigolii* (0–1–1–2 vs. 1–1–0–2, respectively).

In the genus *Neophyllobius*, few descriptions are available for immature stages, of which most are poor, which makes it further difficult to understand the setal ontology. Paredes–León et al. [6] studied the idiosomal and leg setal ontogeny for the species *N*. *cibyci*. It was reported that on the ventral idiosoma, the intercoxal seta *3a* is present in larval stages where the setae *4a* appear in protonymphs and aggenital setae (*ag*) is only present in adult females with one pair of genital (*g*) setae. The redefinition of the family Camerobiidae provided by Fan and Walter [3], with notes on idiosomal chaetotaxy, also stated setae *4a*, *ag* and *g* present in females.

In its support, the species of closely related genus of *Neophyllobius*, the *Tycherobius* (other than the exception mentioned above) have similar situation as that presented in VIC#1. The females of the other two genera, *Camerobia* (Type genus; six species) and *Acamerobia* (one species) also follow the similar ventral idiosoma setal notation.

**Figure 26:**
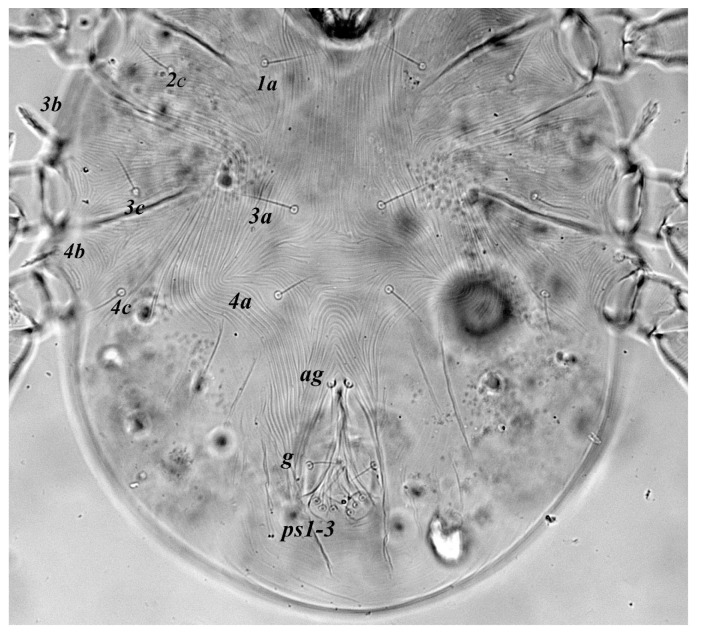
*Camerobia southcotti*. Female. Venter.

**Figure 27:**
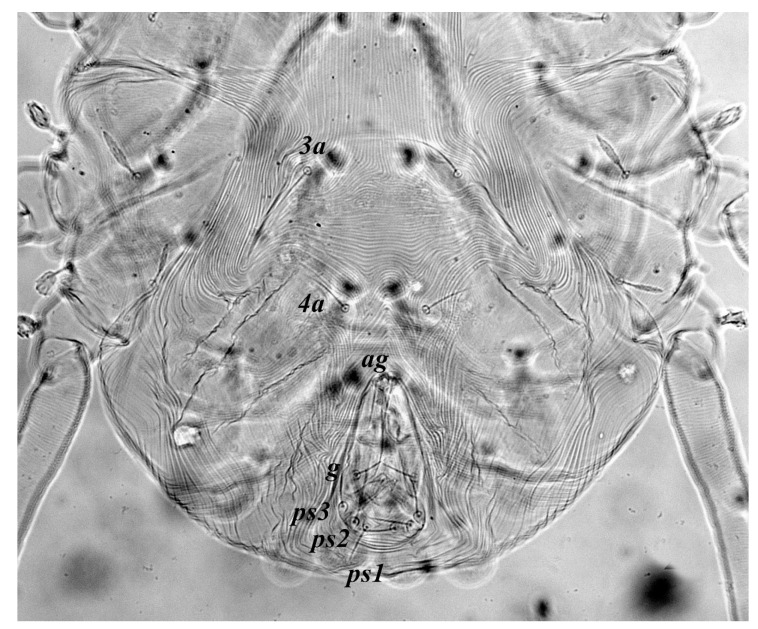
*Decaphyllobius gersoni*. Female. Venter.

**Figure 28:**
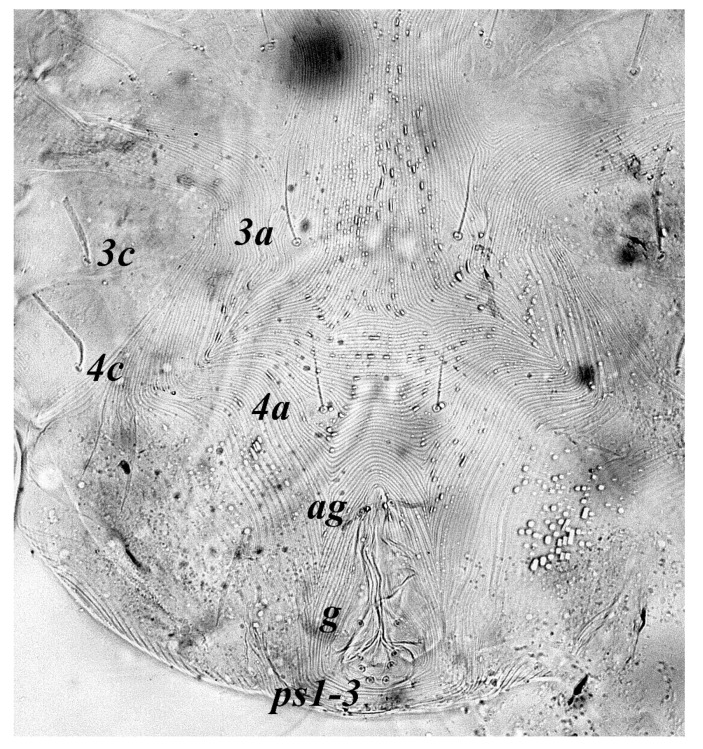
*Neophyllobius lorestanicus* Female. Venter.

**Figure 29:**
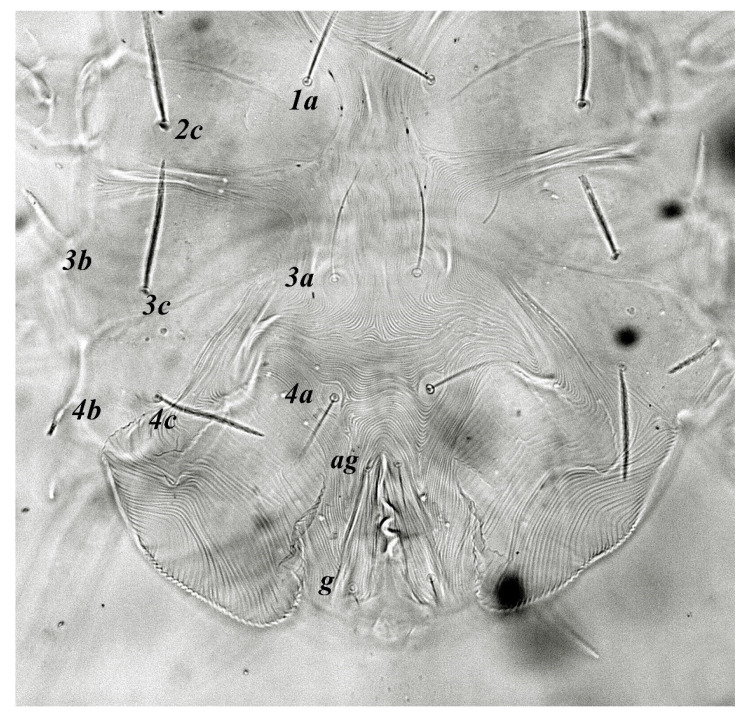
*Neophyllobius denizliensis*. Female. Venter.

**Figure 30:**
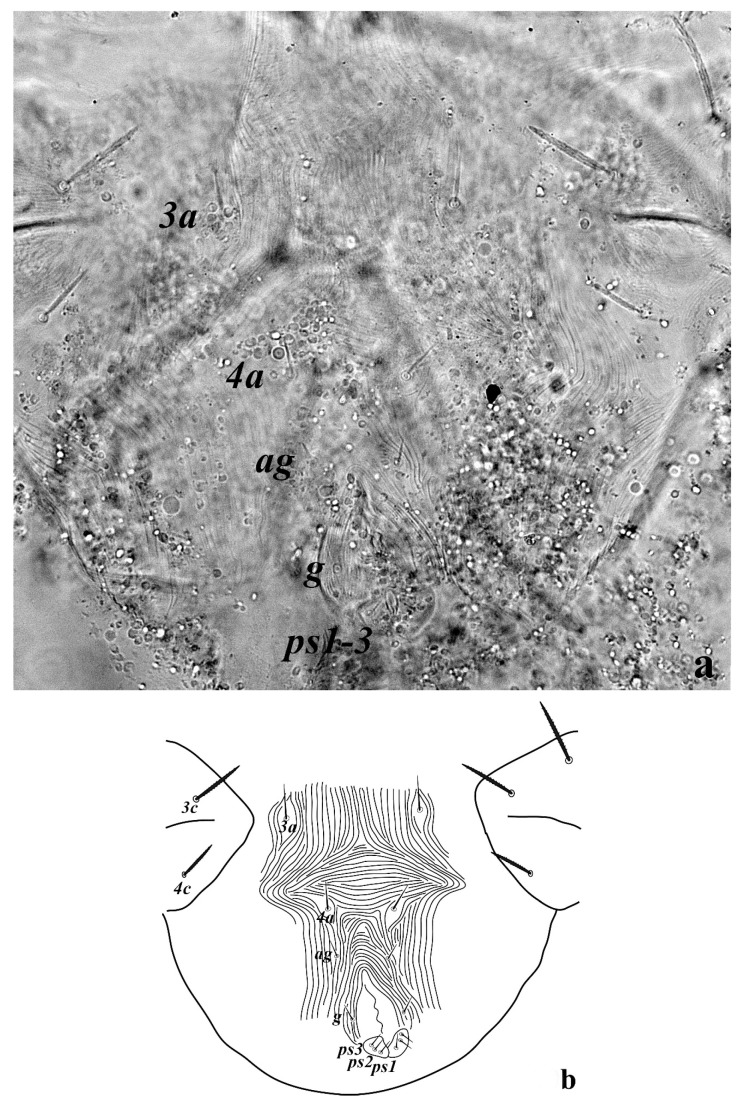
(**a**,**b**) *Tycherobius emadi*. Female. Venter.

In the present and previous studies from Saudi Arabia [26,27], eight species from four genera, viz; *Camerobia*, *Decaphyllobius*, *Neophyllobius* and *Tycherobius* have been reported. As a case study, the ventral idiosoma of all these species are presented (Figure 4, Figure 12, Figure 20, Figure 26,Figure 27,Figure 28,Figure 29,Figure 30a,b). Consistent in females of these species, was the presence of four pairs of ventral setae, excluding 1a, and three pairs of anal setae. The longitudinal striations were present from the level of coxa I till coxa IV. The first pair of ventral setae, present on small platelets, appears in between the coxal setae *3b* and *3c*, where longitudinal striations curve to become transverse. The second pair of ventral setae, also present on small platelets, appears after that distinct transverse striation pattern, more or less at the level of coxal seta *4c*. The position of third pair of setae was variable. Although always anterior to the genital shield, this setal pair was found on or off the anterior margin of genital shield (Figure 4, Figure 12, Figure 20, Figure 26,Figure 27,Figure 28,Figure 29,Figure 30a,b). The fourth pair of setae was always found on the flaps of genital shield.

Based on the understanding from all the published literature on the family Camerobiidae and after carefully observing the relative position of ventral setae in Saudi Arabian camerobiid species, we reach the conclusion that four pairs of setae were present on the ventral idiosoma, excluding setae 1a and three pairs of anal setae (*ps1*–*3*); which were two pairs of intercoxal setae *3a*–*4a* always present on small platelets, a pair of aggenital seta (*ag*), present on ventral integument and on or off the anterior margin of genital shield and a pair of genital setae (*g*) present on sides of genital opening. We support this notation based on the evidence discussed above and recommend future works to follow it.

### 3.11. Key to World Species of the Genus Neophyllobius (Modified after Bolland 1991)

1 Leg tarsi III–IV always with two midventral setae………………new subgenus *Neophyllobius*……15
1‘Leg tarsus III with one or two and tarsus IV always with one midventral seta ………………new subgenus *Monophyllobius*.……2
2 Tarsi III–IV with one midventral ……………………………………………*N*. *variegata* Fan and Walter
2‘Tarsus III with two and tarsus IV with one midventral setae…………………………………………3
3 Femur I–III with 4–3–2 setae ……………………………………………………………………………..4
3‘ Femur I–III with 3–2–1 setae ……………………………………………………………………………14
4 Femur IV with one seta ………………………………………………………………………*N*. *orhani* Doğan and Ayyildiz
4‘ Femur IV with two setae ………………………………………………………………………………5
5 Dorsal striations typically hooked between *c1*–*d1* and *d1*–*e1* …………………………………………….*N*. *interruptus* Bolland
5‘ Dorsal striations not hooked between *c1*–*d1* and *d1*–*e1*………………………………………………6
6 Dorsal seta *d1* the longest setae ……………………………………………………………………*N*. *dichantii* Bolland
6‘ Dorsal seta *d1* not the longest setae ………………………………………………………………………………7
7 Dorsal setae, both *e1* and *f1*, the longest setae …………………………………………………………………8
7‘ Either of the dorsal setae *e1* or *f1* the longest setae ………………….………………………………………………9
8 Tarsus II with 10(*ω*) setae ………………………………………………………………………*N*. *yunusi* Bolland *
8‘ Tarsus II with 9(*ω*) setae …………………………………………………………*N*. *fani*
9 Dorsal setae *e1* the longest setae ………………………………………………………………10
9‘ Dorsal setae *f1* the longest setae ………………………………………………………………11
10 Genu I–II with 1–1 tactile setae, setae *pdx* 10 µm long …….…………………………………………….*N*. *panici* Bolland
10‘ Genu I–II with 2–2 tactile setae, setae *pdx* 57 µm long….……………………………………………………*N*. *mamaneae* Bolland and Swift
11 Genu IV setae two times the genu length …………………………………………………*N*. *texanus* McGregor
11‘ Genu IV setae more than two times the genu length ………………………….………………………………………………………………12
12 Dorsal setae ***d1*** longer than setae *h1* ………………………….………………………………………………………………*N*. *muscantribii*
12‘ Dorsal setae ***d1shorter*** than setae *h1* ………………………….………………………………………………………………………………...13
13 Five pairs of dorso–central setae, *pdx* absent, second seta on femur II is the shortest …………………………………………… *N*. *quinquepilis* Bolland
13‘ Six pairs of dorso–central setae, *pdx* present, second seta on femur II is the longest …………………………………………………*N*. *graminicola* Bolland
14 Dorsal setae *d1* set on strong tubercles and much longer than *e1* and *f1*, setae *h1* based close to *f1* …………………………………*N*. *bialagorensis* Bolland
14‘ Dorsal setae *d1* not on strong tubercles and equal to *e1* and *f1*, setae *h1* based far from *f1* …………………………………………*N*. *vandebundi* Bolland
15 Coxae II with two setae……………………………………….…………………………………………………………………..............……16
15‘ Coxae II with one seta ……………………………………………………………………………….....................……………………………...17
16 Coxae III–IV each with two setae; genu I–II setae long, reaching half the length of respective tibiae ………………………*N*. *bisetalis* Bolland and Swift **+**
16‘ Coxae III–IV each with one setae; genu I–II setae short, less than half the length of respective tibiae ……………………………*N*. *spatulus* De Leon **+**
17 Femur I with 5 setae …………………………………………………………………………………………………*N*. *gonzali* Zaher and Gomaa
17‘ Femur I with 3 or 4 setae………………………………………………………………………………………………………………................. 18
18 Femur I with 3 setae ……..….….. ………………………………………………………………………………*N*. *crinitus* du Toit, Theron and Ueckermann
18‘ Femur I with 4 setae …………………………………………………………………………………………………………....................……… 19
19 Femur II with 4 setae ……………………………………………………………………………………………………………….................... …20
19‘ Femur II with 3 setae …………………………………………………………………………………….....................................................…21
20 All dorsal body setae reaching base of setae in line, dorso–central setae *d1* reaching base of *e1* ………………………………*N*. *quadrisetosus* De Leon
20‘ All dorsal body setae very long, extending beyond the base of setae next in line, dorso–central setae d1 reaching base of *h1* …… *N*. *sultanensis* Akyol and Koç
21 Femur III with 3 setae ………………………………………………………………………………………………………………..............…22
21‘ Femur III with 2 setae ………………………………………………………………………………………………………………..............…33
22 Femur IV with 1 or 2 setae ………………………………………………………………………………………………………………..............23
22‘ Femur IV with 3 setae ………………………………………………………………………………………………………………..............…24
23 Femur IV with 1 seta ……………………………………………………………………………………………………………*N*. *foliosetosus* Fan
23‘ Femur IV with 2 setae ………………………………………………………………………………………………………………..............…25
24 Lateral setae vi long, at least two times of h2, two most proximal setae on femur III on one level ……………………………………………………….... *N*. *ueckermanni* Bolland
24‘ Lateral setae vi normal, shorter than two times of h2, two most proximal setae on femur III not on one level …………………………………………. *N*. *sanctaeluciae* Bolland
25 Dorso–central setae longer than interval to setae next behind …..................... ………………………………26
25‘ Dorso–central setae just reach or shorter than interval to setae next behind .. …………………………………………29
26 Dorsal setae *d1* longest setae …………………………….………………………*N*. *trisetosus* Bolland
26‘ Dorsal setae *e1* longest setae ………………………………………………………………………………………………………………..............…27
27 Dorsal setae *d1* longer than *c1*, coxal setae different in length ………………. 28
27‘ Dorsal setae *d1* as long as *c1*, coxal setae equal in length … *N*. *montanus* Bolland
28 Genu II and III setae as long as genu, palps small ………. *N*. *capparidis* Bolland
28‘ Genu II and III setae longer than genu, palps thicker …… *N*. *graminum* Bolland
29 Dorso–central setae reaching to setae next in line …….. *N*. *glaesus* Zmudzinski
29‘ Dorso–central setae shorter than the distance between setae next in line....... 30
30 Some dorso–central setae shorter than interval to setae next behind ……….. 31
30‘ All dorso–central setae shorter than interval to setae next behind ………………………………………………………….... *N*. *bequartiodendri* Bolland
31 Genu IV seta five times longer than genu and longer than half the length of tibia IV ………………………………………………………………. *N*. *mkuzensis* du Toit et al.
31‘ Genu IV seta twice as long as genu and less than half the length of tibia IV.. 32
32 Dorsal setae ***c1*** and ***d1*** shorter than interval to setae next behind, third and fourth seta on femur I equal in length, distal seta on palpfemur at least two times longer than the proximal seta, coxa I setae nearly equal in length ………… *N*. *gigantorum* Bolland
32‘ Dorsal setae ***c1*** shorter and ***d1*** longer than interval to setae next behind, third seta on femur I much shorter than fourth seta, distal seta on palpfemur not two times longer than the proximal seta, coxa I setae much different in length *N*. *hypoleanae* Bolland
33 Femur IV with 2 setae …………………………………………………… 34
33‘ Femur IV with 1 seta ………………………………………………….............. 122
34 Palptarsus with 1 eupathidion ………………………………………………….. 35
34‘ Palptarsus with 2 eupathidia ……………………………………………………. 37
35 Palptarsus with 1 seta ………………………….… *N*. *euonymi* Bolland and Ripka
35‘ Palptarsus with 2 setae …………………………………………………………… 36
36 Dorso–central setae *d1*, *e1*, *f1* equal in length ………………. *N*. *plumifer* Bolland
36‘ Dorso–central setae *d1* the longest dorsal setae ……*N*. *demirsoyi* Akyol and Koç
37 Palptarsus with 3 setae …………………............................................................... 38
37‘ Palptarsus with 2 setae …………………………………………………………… 42
38 Genu I–II, each with 1 solenidion ……………………………………………….. 39
38‘ Genu without solenidia ………………….…. α *N*. *edwardi* Khanjani and Hoseini
39 Tibiae II with 9 tactile setae …………………………… *N*. *zolfigolii* Khanjani et al.
39‘ Tibiae II with 8 tactile setae …………………………………………….………. 40
40 Dorso–central setae *c1* and *d1* equal in length .. *N*. *dogani* Khanjani and Hoseini
41‘ Dorso–central seta *d1* longer than *c1* ………........................................................ 41
41 Tarsi I–II with 9–8 tactile setae ……………….. *N*. *seemani* Khanjani and Hoseini
41‘ Tarsi I–II with 10–9 tactile setae ………………………… *N*. *mitrae* Khanjani et al.
42 Band of coarse striae interrupted and hooked between setae *c1* and *d1* …………………………………..................................................... *N*. *natalensis* Meyer and Ryke
42‘ Striae neither interrupted nor hooked between setae *c1* and *d1 .*…………….. 43
43 Two solenidia on distal end of tibia I, one solenidion on the distal end of tibiae II–IV …………………………………………………………………………………………….. 44
43‘ One solenidion on distal end of tibiae I–IV ……………………………………. 46
44 Tarsus II with 9 tactile setae …………………………. *N*. *kamalii* Khanjani et al. *
44‘ Tarsus II with 10 tactile setae ……………………………………………………. 45
45 In males, genu I seta less than fifth the length of tibiae I, coxae I–IV without polygonal dimples …………………………………………. *N*. *karabagiensis* Akyol and Koç *
45‘ In males, genu I seta less than third the length of tibiae I, coxae I–IV with polygonal dimples …………………………………………… *N*. *sycomorus* Zaher and Gomaa β
46 Dorsal setae *c1* long, passes at least bases of *e1* ……………………………….. 47
46‘ Dorsal setae *c1* just reaching or shorter than the distance to bases of *e1 ..*....... 58
47 Dorsal setae *e1* longer than *c1* ……………………………………………………. 48
47‘ Dorsal setae *e1* as long as or shorter than *c1* …..……………………………….. 52
48 Dorsal setae *c1* longer than *d1* …………… *N*. *nemoralis* Kuznetsov and Livshits
48‘ Dorsal setae *c1* shorter than *d1* …………………………………………………... 49
49 Setae *h1* longer than *h2* …………………………………………………………… 51
49‘ Setae *h1* equal to or shorter than setae *h2* ………………………………………. 50
50 Tarsus II with 9 tactile setae, dorso–central setae *c1*, *d1*, *e1* very long > 200 µm in length …………………………………………… *N*. *astragalusi* Khanjani and Ueckermann
50‘ Tarsus II with 10 tactile setae, dorso–central setae *c1*, *d1*, *e1* < 200 µm in length ……………………………………………………………….. *N*. *platanobius* Bolland
51 Tarsus II with 10 setae, tibiae III with 7 setae, setae e1 the longest…………………………………………………………………………… *N*. *podocarpi* Bolland
51‘ Tarsus II with 9 setae, tibiae III with 8 setae, setae d1 and e1 almost same in length ………………………………………………………………………... *N*. *izmirensis* Akyol
52 Setae *e1* as long as setae *c1* ………………………………. *N*. *parthenocissi* Bolland
52‘ Setae *e1* shorter than setae *c1*…………………………………………………….. 53
53 Dorsal setae very small, distal seta on femur I not reaching femur–genu boarder …………………………………………………………………………………………. 57
53‘ Dorsal setae thicker, distal seta on femur I easily reaching femur–genu boarder ………………............................................................................................................ 54
54 Dorsal setae *d1* and *e1* unequal in length ………………………………………. 55
54‘ Dorsal setae *d1* equal in length to setae *e1* …………………. *N*. *femoralis* Bolland
55 Tarsus II with 10 tactile setae ……………………... *N*. *turcicus* Koç and Ayyildiz
55‘ Tarsus II with 9 tactile setae …………………………………………………….. 56
56 Dorso–central setae *pdx*, unpaired, single, 75 µm long ……………………………………………….….. *N*. *ostovani* Khanjani and Hoseini
56‘ Dorso–central setae *pdx*, paired, 88 µm long…………………………………………………*N*. *asalii* Khanjani and Ueckermann
57 Dorso–central setae *c1* not reaching the base of setae *f1* … *N*. *tenuipilis* Bolland
57‘ Dorso–central setae *c1* long, reaching the base of setae *f1 .*………………………………………………………… *N*. *afyonensis* Akyol and Koç
58 Setae *d1* reach or pass bases of setae *f1* …………………………………………. 59
58‘ Setae *d1* do not reach at all to bases of setae *f1* …………………………………. 75
59 Setae *e1* do not reach setae *h2* ……………………………………………………. 60
59‘ Setae *e1* reach or extend beyond the setae *h2* ………………………………….. 70
60 Setae *e1* reach bases of *h1* ……………………………............................................. 61
60‘ Setae *e1* does not reach the bases of *h1* ……………............................................. 63
61 Genu I–II setae long, extending beyond half the length of respective tibiae ………………………………………………………….. *N*. *populus* Akyol and Koç
61‘ Genu I–II setae short, reaching less than half the length of respective tibiae .. 62
62 Dorsal setae *c1* longer than *pdx* ………………. *N*. *mangiferus* Zaher and Gomaa
62‘ Dorsal setae *pdx* and *c1* equal in length ………….……… *N*. *theobromae* Bolland
63 Setae *f1* pass easily the bases of setae *h1* ………….……………………………. 64
63‘ Setae *f1* just reach the bases of setae *h1* …………………. *N*. *marginatus* De Leon
64 Setae *c1* easily reach bases of setae *d1* ………………………………………….. 66
64‘ Setae *c1* and *pdx* do not reach bases of setae *d1* ……………………………….. 65
65 Leg tarsi I–III with 10–10–8 tactile setae, genu IV seta not reaching tibial border ………………………………………………………………….. *N*. *longulus* De Leon
65‘ Leg tarsi I–III with 9–9–7 tactile setae, genu IV seta long, reaching tibial border …………………………………………. *N*. *persiaensis* Khanjani and Ueckermann
66 Setae *pdx* reach bases of setae *d1* ………………………………………………… 68
66‘ Setae *pdx* do not reach bases of setae *d1* ………………………………………… 67
67 Second seta on the femur II the longest, palptarsus without solenidion …………………........................................................................ *N*. *hispanicus* Bolland
67‘ First and second setae on femur II equal in length, palptarsus with one solenidion ……………………………………………………………………….. *N*. *denizlyensis* Akyol
68 Genu I–III setae not whip like, not extending beyond half the length of corresponding tibiae ……………………………………………………………………………….. 69
68‘ Genu I–III setae whip like, extending till corresponding tarsi border …………………………………………………….. *N*. *bolvadinensis* Akyol and Koç
69 Setae *d1* the longest dorsal body setae ……..………………… *N*. *deleoni* Bolland
69‘ Setae *c2* the longest dorsal body setae.. *N*. *helichrysi* du Toit, Theron and Ueckermann
70 Most distal seta on femur I longer than the third one ………………………….71
70‘ Most distal seta on femur I shorter than the third one …… *N*. *communis* Gerson
71 Setae *c1* and *pdx* shorter than *f1* …………………………………………………. 72
71‘ Setae *c1* longer than *f1* ……………………………………………………………. 73
72 Tarsus II with 9 tactile setae ………………………. *N*. *lorestanicus* Khanjani et al.
72‘ Tarsus II with 10 tactile setae …………………………….. *N*. *lachishensis* Bolland
73 Dorso–central seta *c1*, *d1* and *e1* not equal in length ………………………….. 74
73‘ Dorso–central setae *c1*, *d1* and *e1* equal in length ………………………………………………… *N*. *pistaciae* Bolland and Mehrnejad
74 Tarsi I–II with 10 setae each ………………………………..*N*. *levanticola* Bolland
74‘ Tarsi I–II with 9 setae each …………………………….. *N*. *saberi* Ahaniazad et al.
75 Third seta on femur I shorter than the fourth seta …………………………….. 76
75‘ Third seta on femur I longer than the fourth seta …………………………….. 83
76 Most distal seta on femur I not reaching the genu border, *pdx* reaching the marginal side of the dorsum ……………………………………………………………………… 77
76‘ Most distal seta on femur I passing the genu border, *pdx* not reaching the marginal side of the dorsum ……………………………………………………………………… 78
77 All genu setae shorter than the length of respective leg ………………………………...…………………………*N*. *quercus* Uluçay and Koç
77‘ All genu setae very long, extending beyond the length of respective leg ……………………………………………………………….. *N*. *lamimani* McGregor
78 Most distal seta on femur I about 3–4 times longer than the third seta .......... 79
78‘ Most distal seta on femur I shorter than 3 times the third seta ……………… 82
79 Distal seta on femur II shorter than 1/3 of the length of the proximal one …………………………………………………………………….. *N*. *coxalis* Bolland
79‘ Distal seta on femur II longer than 1/3 of the length of the proximal one ….. 80
80 Dorso–central setae *pdx* and c1 equal in length, shorter than *d1*, *e1* and *f1*….. 81
80‘ Setae *c1* the longest dorso–central setae …….. *N*. *olurensis* Doğan and Ayyildiz
81 Setae *c1* reach half to the distance *c1*–*d1*, third seta on tibia I close to seta one and two, setae *e1* longer than *f1* …………………………………………. *N*. *arenarius* Bolland
81‘ Setae *c1* reach till bases of *d1*, third seta on tibia I far away from seta one and two, setae *e1* as long as *f1* ………………………………………………. *N*. *oregonensis* Bolland
82 Setae *c1* do not reach the bases of *d1*, the distance *e1*–*f1* much shorter than normal ………………………………………………………………………. *N*. *danthoniae* Bolland
82‘ Setae *c1* reach the bases of *d1*, setae *e1* not based close to setae *f1* ……………………………………………………………….. *N*. *coloradensis* Bolland
83 Distal femur I seta passes genu border ….............................................................. 84
83‘ Distal femur I seta just reaches or not reaching the genu border at all ……… 90
84 Third seta of femur I as long as or longer than the fourth seta ……………… 88
84‘ Third seta of femur I equal to or more than half the length of the fourth ……85
85 Setae *d1* longer than *e2* ……………………………………………………………. 86
85‘ Setae *d1* shorter than or as long as *e2* …………………………………………… 87
86 Second seta on femur I shorter than the third one, femur I setae more lanceolate ………………………………………………………………… *N*. *parisianus* Bolland
86‘ Second seta on femur I longer than the third one, femur I setae more whip like …………………………………………………………………*N*. *burrellis* McGregor
87 Setae *c1* do not reach bases of *d1* …………………………. *N*. *ponderosus* Bolland
87‘ Setae *c1* just reach or pass bases of *d1* …………………… ***N***. ***dickansoni*** Bolland
88 Genu I–III setae equal to the length of segment ……………………………..…….………………………………………………*N*. *succineus* Bolland and Magowski
88‘ Genu I–III setae longer than the length of segment …………………………. 89
89 Genu III setae reaching or just passing first row of tibia setae, distal setae on femur III reach genu border ………………………………………… *N*. *sturmerwoodi* Bolland
89‘ Genu III setae longer than tarsus border, distal setae on femur III does not reach genu border ……………………………………………………………………*N*. *aesculi* Bolland
90 None of the dorso–central setae reach bases of the setae next in line, all dorsal and femoral setae short, small and equal in length, genu setae passing tarsus end …………………………………………………………………. *N*. *tamarindi* Bolland
90‘ Some or all dorso–central setae reach bases of the setae next in line …………91
91 Some of the dorso–central setae reach or cross the bases of setae next in line (i.e., *e1* or *e1* and *f1*) ….………………………………………………………………………… 92
91‘ Nearly all dorsal or dorso–central setae reach the bases of setae next in line . 95
92 Setae *e1* and *f1* only longest dorso–central setae ……………………………… 93
92‘ Only setae e1 the longest dorso–central setae ..………………………………... 94
93 Tarsi II with 10(+ω) setae, genu IV setae not reaching base of corresponding tarsi ………………………………………………………………………… *N*. *conocarpi* Bolland
93‘ Tarsi II with 9(+ω) setae, genu IV setae reaching base of corresponding tarsi ………………………………………………………….............*N*. *ayvalikensis* Akyol
94 Femur setae very short, femur I setae 3 and 4 nearly at one level, coxal setae *1c* twice the length of *1b*, genus I seta passes tibiae and genu II and IV seta passing tarsi ends ………………………………………………………..…. *N*. *lorioi* Smiley and Moser
94‘ Femur setae longer, coxal seta *1c* nearly as long as *1b*, none of the genu setae reaching tibia end ……………………………………………………………. *N*. *acaciae* Bolland
95 Femur I–IV strongly serrulate at their proximal posterior margins …………………………………………………………………………………*N*. *lobatus* De Leon
95‘ Femur I–IV not strongly serrulate at their bases ……………………………… 96
96 Femur I setae 3 and 4 not based on one or nearly one level ………………….. 97
96‘ Femur I setae 3 and 4 based on one or nearly one level ……...........................113
97 Genu I–III and femur I–IV setae strongly lanceolate ………………………….. 98
97‘ Genu I–III setae not lanceolate …………………………………………………... 99
98 Distal setae on femur IV not passing genu border, coxal seta *1c* longer than *1b*…………………………………………………………………. *N*. *ceratoniae* Bolland
98‘ Distal setae on femur IV passing genu border, coxal seta *1c* shorter than *1b*…………………………………………………………………*N*. *lanceolatus* Bolland
99 Genu I setae not reaching second row of tibia setae ………………………... 100
99‘ Genu I setae reaching or longer than second row of tibia setae …………….. 104
100 Genu II setae not reaching second row of tibia setae …………………………. 101
100‘ Genu II setae reaching second row of tibia setae ……….. *N*. *binisetosus* Bolland
101 Genu III setae not whip like and plumed at the top ……….. *N*. *atriplicis* Bolland
101‘ Genu III setae whip like …………………………………………………………. 102
102 Genu II setae not reaching the first row of tibiae setae, dorso–central setae *d1*, *e1* and *f1* much longer than other dorsal setae, coxal setae *1c* much shorter than *1b* …… …………………………………………………………………….*N*. *ambulans* Meyer
102‘ Genu II setae reaching at least the first row of tibiae setae …......................... 103
103 Dorso–central setae d1 and e1 much longer than other dorsal setae ……… …………………………………………………. *N*. *cavumarboris* Meyer and Ryke
103‘ Dorso–central setae c1 longer than other dorsal setae …………………………………………….. *N*. *camelli* Khanjani and Ueckermann
104 Genu II setae not whip like ….…………………………………………………. 105
104‘ Genu II setae whip like ………………………………………………………….. 107
105 Proximal setae on femur IV reaching the base of distal setae ......................... 106
105‘ Proximal setae on femur IV not reaching the base of distal setae………………………………………………………………*N*. *armeniaca* Bolland
106 Genu II seta long whiplike, crossing first row of setae on respective tibiae ……………………………………………… *N*. *abiegnus* Khaustov and Abramov
106‘ Genu II seta short, not reaching first row of setae on respective tibiae …………………………………………………..… *N*. *ayyildizi* Koç and Madanlar
107 Genu I and II setae reach till second row of tibia setae ……………………… 108
107‘ Genu I and II setae are passing tibial border …………………………………. 109
108 Dorsal setae small, not pointed, body small, coxa 1 seta *1c*:*1b* = 6:18 …………………………………………………………………… *N*. *iramus* De Leon
108‘ Dorsal setae more broad and occupied with very strong stings, coxa I seta *1c*:*1b* = 6:17 ………………………………………………………………………… *N*. *equalis* De Leon
109 Dorsal setae specially spinosed ………………………………………………………………………… *N*. *setosus* Bolland
109‘ Dorsal setae not specially spinosed ………………………………………………………………………… 110
110 First seta on femur II not reaching the third, proximal femur I seta not the longest ……………………………………………………………................................................. 111
110‘ First seta on the femur II the longest femur seta which passes easily the third, proximal femur I seta the longest …………………………………………… *N*. *pruni* Bolland
111 Setae *e1* longest among the dorso–central setae……………………………………
……………………………………………………*N*. *askalensis* Doğan and Ayyildız
111‘ Setae *e1* not the longest among the dorso–central setae …………………….. 112
112 Proximal seta on femur I equal in length with the second, distal seta on femur IV long, passes genu border, first seta on femur I reaches the bases of the fourth ……................................................................................................. *N*. *viticola* Bolland
112‘ Proximal seta on femur I shorter than the second, distal seta on femur IV short, not passing genu border, first seta on femur I does not reach the fourth seta at all ………………………………………………………………………… *N*. *combreticola*
113 Dorsal setae broad, setae d1 strong, genu II seta as long as genu, genu I and II setae not reaching first row of tibiae setae ………………………………………………… 114
113‘ Dorsal setae smaller, genu I and II setae at least reaching first row of tibiae setae ……………………………………………………………………………………... 115
114 Genual setae distinctly spinose …………………………….. *N*. *curtipilis* De Leon
114‘ Genual setae linear and faintly spinose …………………….. *N*. *spatulus* De Leon
115 Genu I setae reach till first row of tibiae setae, palp femur swollen, palp genu short ………………………………………………………………………… *N*. *sierrae* McGregor
115‘ Genu I setae longer, palp femur longer ……………………………………… 116
116 Two eupathidia on the palp tarsus based very different in level, dorsal setae small with many spicules ……………………………………………….. *N*. *americanus* Banks
116‘ Two eupathidia on the palp tarsus on similar level, dorsal setae broader with less spicules …………………………………………………………………………………... 117
117 Genu III–IV setae are passing tarsus end ……………………………………… 118
117‘ Genu III–IV setae not reaching beyond end of respective legs ……………… 120
118 Dorso–central setae *pdx* and *c1* grouped on a small finely striated platelet ……………………………………………………. *N*. *tescalicola* Parades–Leon et al.
118‘ Dorso–central setae *pdx* and *c1* not grouped on a platelet …………………… 119
119 Lengths of dorsal setae *c1* and *d1* are the same as the distance between setae *c1*–*d1* and *d1*–*e1* respectively ………………………………………………. *N*. *farrieri* De Leon
119‘ Dorsal setae *c1* and *d1* are distinctly longer than the distance between setae *c1*–*d1* and *d1*–*e1* respectively ……………………………………… *N*. *cibyci* Parades–Leon et al.
120 Setae *sce* shorter *sci* ……………………………………………………… 121
120‘ Setae *sce* equal in length to *sci* …………………………Ω*N*. *consobrinus* De Leon
121 Lateral setae *vi*, *ve* and *sci* subequal in length ……………Ω*N*. *inequalis* De Leon
121‘ Lateral setae sci distinctly longer than *vi* and *ve* ………… Ω*N*. *piniphilus* Bolland
122 Setae *e1* passes the bases of *h1* ………….……. ***N***. ***aegyptium*** Soliman and Zaher
122‘ Setae *e1* do not reach the bases of *h1* ……………………………………… 123
123 Genu I seta short not reaching second row of setae on corresponding tibiae ……………………………………… 124
123‘ Genu I seta long, extend beyond corresponding tarsus base ……………… 126
Tarsi I–II with 8 setae each ……………………………………*N*. *ferrugineus* Fan
124‘ Tarsi I–II with 10 setae each ……………………………………… 125
125 Coxal seta *1c* twice as long as coxal seta *1b* …. *N*. *lalbaghensis* Zeity and Gowda
125‘ Coxal seta *1c* 1.5 times as long as coxal seta *1b* … *N*. *womersleyi* Fan and Walter
126 Distance *e1*–*f1* two times longer as distance *d1*–*e1*, setae *f1* the longest ………………………………………………………………… *N*. *niloticus* Bolland
126‘ Distance *e1*–*f1* sub–equal to *d1*–*e1* ……………………………………………. 127
127 Palp femur with both setae short, not reaching end of palp genu; long setae of genu I–III not reaching end of respected legs …………………. *N*. *bamiensis* Khanjani et al.
127‘ Palp femur with one of two setae reaches end of tarsus ……………………. 128
128 Dorsal setae *d1* reaching base of *e1*, coxal setae *1c* longer than *2c* in length ………………………………………………………………………………... *N*. *fissus*
128‘ Dorsal setae *d1* extend beyond the bases of *e1*, coxal setae *1c* almost equal to *2c* in length ……………………………………………………………………*N*. *punctulatus* Fan
* = species described based on male holotype.
+ = two species, viz; *N*. *bisetalis* and *N*. *spatulus* with two setae on coxae II as mentioned by Bolland and Swift [50] and Bolland [16], respectively.
α = The character of genu I–IV without solenidion is mentioned in the original description [38].
β = The male specimens were not reported at the time of original description for ***N***. *sycomorus*. However, Bolland [16] provided very few morphological characters of *N*. *sycomorus* with the illustrations of male and female.
Ω = These three species have minute differences among them. Bolland and Swift [50] have also questioned the close similarity of *N*. *consobrinus* and *N*. *inequalis* suggesting later could be a deutonymph of former. However, these species are placed in the diagnostic key based on available information. but types of each species require re–examination.

## Figures and Tables

**Table 1:** Morphological characters (excluding solenidion) of the three genera (after Fan & Walter, 2011 [3]; Uluçay et al., 2016 [15]; Nasrollahi et al., 2019 [23]).

		*Monobius* gen. nov. (2 Species)	*Tycherobius* (26 Species)	*Neophyllobius*	
				*Neophyllobius* (114)	*Monophyllobius* (15)
Coxa I		2–3	2–3	2–3	3
Coxa II		1	1	1–2	1
Coxa III		2	2	1–2	2
Coxa IV		2	1–2	1–2	2
Femur I		4	3–4	3–5	3–4
Femur II		3	3	2–4	2–3
Femur III		2–3	1–4	1–3	1–2
Femur IV		2	1–3	1–3	1–2
Genu I		1	1	1–2	1–2
Genu II		1	1	1–2	1–2
Genu III		1	1	1	1
Genu IV		1	1	1	1
Tibiae I		9	8–9	8–10	8–10
Tibiae II		8	7–8	7–9	7–9
Tibiae III		8	6–8	7–9	7–9
Tibiae IV		7	6–7	6–8	6–8
Tarsus I		9	7 or 9 or 10	7–11	10–11
Tarsus II		9	7–10	6/8–11	9–10
Tarsus III		7	7	6–8	7–8
Tarsus IV		7	7	7–8	7
Midventral setae on tarsi I–IV	Number	1–1–1–1	2–2–1–1	2–2–2–2	2–2–2(1)–1
	Position	–	not in a longitudinal line, variously spaced	in a longitudinal line	

**Table 2:** Morphological characters of *Neophyllobius* species with incomplete information.

Species		*vanderwieli*	*ornatus*		*mexicanus*	*summersi*	*saxatilis*	*elegans*	*guajavae*	*hyderabadensis*	Species 1	Species 2
**Published Year**		1926	1940		1950	1950	1938	1886	2002	1980	2006	2005
**Country**		Netherland	Australia		Mexico	California	Ireland	Italy	India	India	Yemen	Hungary
**Habitat/Host plant**		Nest of *Talpa europaea*.	Apiomorpha galls on *Eucalyptus* sp.	–	*Zanthoxylon* sp.	Saltgrass	Lichen covered rocks	–	*Psidium guajava*	*Caryota urens*	Malaise Trap	D–Vac Sample
**Body**	**Length**	–	250	250	–	–	320	250	240	No Literature/Description Available		
	**Width**	–	175	175	–	–	210	220	178			
**Number of dorsal setae**	**mc**	6	6 (?)	–	15?	6	6	6	6			
	**l**	9	9 (?)	–		9	9	9	9			
**Number of setae on leg segments**	**coxae**	3–1–2–2	3–1–?–?	1a+2–1–?–?	–		3–1–2–2	3–1–2–2	–			
	**trochanter**	1–1–1–1	1–1–1–1	1–1–1–1	–	1–1–1–1	1–1–1–1	1–1–1–1	–			
	**femora**	4–3–2–2	4–2?–2?–1?	4–2?–2?–1?	–	4–––	3?–1?–2–2	4–3–2–2	–			
	**genua**	1–1–1–1	1–1–1–1	1–1–1–1	–	1–1–1–1	1–1–1–1	1–1–1–1	–			
	**tibiae**	9–8–8–7	?–8–8–7	?–8–8–7	–	–	?–8–8–7	9–8–8–7	–			
	**tarsi**	10–10–8–8	2–2–2–2	2–2–2–2	–	2–––	?–10–8–8	10–10–8–8	–			
**Number of setae on palp**	**trochanter**	0	–	0	–	–	0	0	–			
	**femora**	2	2	2	–	3	2	2	–			
	**genua**	1	2	2	–	–	1	1	–			
	**tibiae**	3+1 sword like	2?+1 sword like	2+1 claw	–	–	3+1 sword like	?+?	–			
	**tarsi**	2+2 eup	2+2 eup	2+2 eup	–	–	2+2 eup	?+2 eup	–			
**Reference**		[16]	[16]	[3]	[10,16]	[10,16]	[16]	[16]	[41]	[7]	[48]	[49]

“?” = the original author was not sure of the information and provided no specific details. “–” = unavailable.

**Table 3:** Ventral idiosoma setal notation described and illustrated in 55 species of *Neophyllobius*.

Species	Ventral Idiosomal Setae				Species	Ventral Idiosomal Setae			
	*3a*	*4a*	*ag*	*g*		*3a*	*4a*	*ag*	*g*
*abiegnus*	1	1	1	1	*lamimani*	1	1	1	1
*afyonensis*	1	1	1	1	*lorestanicus*	0	1	1	2
*asalii*	0	1	1	2	*mamaneae*	1	1	1	1
*askalensis*	1	1	0	2	*mangiferus*	1	1	0	2
*astragalusi*	1	0	1	2	*mitrae*	0	1	1	2
*ayvalikensis*	1	0	1	2	*olurensis*	1	1	0	2
*ayyildizi*	1	0	1	2	*orhani*	1	1	0	2
*bamiensis*	0	1	1	2	*ostovani*	0	1	1	2
*bisetalis*	1	1	1	1	*parisianus*	1	0	1	2
*bolvadinensis*	1	0	1	2	*parthenocissi*	1	1	1	1
*camelli*	1	0	1	2	*persiaensis*	1	0	1	2
*cibyci*	1	1	1	1	*pistaciae*	1	0	1	2
*communis*	1	1	0	2	*podocarpi*	1	1	0	2
*consobrinus*	1	1	1	1	*populus*	1	1	0	2
*crinitus*	1	0	1	2	*punctulatus*	1	1	0	2
*demirsoyi*	1	0	1	2	*quercus*	1	1	1	1
*denizliensis*	1	1	1	1	*saberi*	0	0	2	2
*dogani*	0	1	1	2	*saxatilis*	1	1	–	–
*edwardi*	0	1	1	2	*seemani*	0	1	1	2
*euonymi*	1	0	1	2	*sturmerwoodi*	1	1	1	1
*fani*	1	1	0	2	*sultanensis*	1	1	1	1
*ferrugineus*	1	1	0	2	*sycomorus*	1	1	0	2
*foliosetosus*	1	1	0	2	*tepoztalensis*	1	1	1	1
*gonzali*	1	1	0	2	*tescalicola*	1	1	1	1
*izmirensis*	1	0	1	2	*womersleyi*	1	1	1	1
*karabagiensis*	1	1	1	1	*yunusi*	1	0	1	2
*lachishensis*	1	0	1	2	*zolfigolii*	0	1	1	2
*lalbaghensis*	1	1	1	1					

*N*. *bamiensis*: illustration depicts presence of paired *3a*, *4a*, *ag* and *g* setae. *N*. *saberi*: illustration depicts presence of paired *4a*, *ag* and *g1*–*2* setae *N*. *zolfigolii*: illustration depicts presence of paired *3a*, *4a* and *g1*–*2.*

## Data Availability

This published work and the nomenclatural acts it contains have been registered in ZooBank, the online reg-istration system for the ICZN (International Code of Zoological Nomenclature).
